# Thermal titration molecular dynamics (TTMD): shedding light on the stability of RNA-small molecule complexes

**DOI:** 10.3389/fmolb.2023.1294543

**Published:** 2023-11-13

**Authors:** Andrea Dodaro, Matteo Pavan, Silvia Menin, Veronica Salmaso, Mattia Sturlese, Stefano Moro

**Affiliations:** Molecular Modeling Section (MMS), Department of Pharmaceutical and Pharmacological Sciences, University of Padova, Padova, Italy

**Keywords:** ligand-RNA complex, thermal titration molecular dynamics, TTMD, molecular docking, molecular dynamics, interaction fingerprints

## Abstract

Ribonucleic acids are gradually becoming relevant players among putative drug targets, thanks to the increasing amount of structural data exploitable for the rational design of selective and potent binders that can modulate their activity. Mainly, this information allows employing different computational techniques for predicting how well would a ribonucleic-targeting agent fit within the active site of its target macromolecule. Due to some intrinsic peculiarities of complexes involving nucleic acids, such as structural plasticity, surface charge distribution, and solvent-mediated interactions, the application of routinely adopted methodologies like molecular docking is challenged by scoring inaccuracies, while more physically rigorous methods such as molecular dynamics require long simulation times which hamper their conformational sampling capabilities. In the present work, we present the first application of Thermal Titration Molecular Dynamics (TTMD), a recently developed method for the qualitative estimation of unbinding kinetics, to characterize RNA-ligand complexes. In this article, we explored its applicability as a post-docking refinement tool on RNA in complex with small molecules, highlighting the capability of this method to identify the native binding mode among a set of decoys across various pharmaceutically relevant test cases.

## 1 Introduction

Following the “Central Dogma” of molecular biology, RNA has been historically perceived as a bridging element between DNA genetic information and protein biosynthesis (Bissaro et al., 2020; Pavan et al., 2022a). However, this paradigm shifted in the last decades due to discoveries that showcased RNA’s strikingly complex genetic and catalytic functions (Sponer et al., 2018; Childs-Disney et al., 2022). Therefore, this biomolecule is no longer considered just a carrier of information. Instead, it is perceived as one of the most pluripotent actors in molecular biology (Sponer et al., 2018).

Considering that only a fraction (3%) of the genome is translated into proteins (Zafferani and Hargrove, 2021) non-coding RNAs (ncRNAs) are under the spotlight as they are involved in epigenetics (Falese et al., 2021; Childs-Disney et al., 2022), playing a pivotal role in the etiopathogenesis of both cancer and neurodegenerative diseases (Falese et al., 2021).

The identification of RNAs as possible therapeutic targets and the increased availability of experimentally solved three-dimensional structures of RNA complexes paves the way for the application of structure-based drug design (SBDD) techniques to characterize the interaction between small molecules and RNA, allowing the possibility of rationally discover new hits and steer their development into mature leads (Pavan et al., 2022a; Childs-Disney et al., 2022). Although it is an appealing perspective, applying routinely adopted molecular modeling protocols to RNA systems is not trivial. Historically, these techniques have been optimized to study the recognition process between small organic molecules as ligands and proteins as receptors (Salmaso and Moro, 2018; Bassani and Moro, 2023; Pavan and Moro, 2023). Nevertheless, structural differences between proteins and RNAs, such as the peculiar surface charge properties portrayed by the polyanionic phosphate backbone, the ions’ role in the structural stability and folding of RNA, the role of the solvent in mediating structural stability and forming bridged interactions, other than the intrinsic structural flexibility of ribonucleic acids, limited so far the possibility to repurpose these methodologies to the study of RNA complexes (Bissaro et al., 2020).

So far, different computational techniques have been developed to tackle the study of RNA-small molecule complexes from different perspectives, from binding site identification to binding mode prediction, scoring, and characterization of time-dependent properties (Zhou et al., 2022).

One of the most successful approaches in structure-based drug design is molecular docking, a fast and reliable protocol that provides possible binding hypotheses generated through a conformational search algorithm and ranked through a scoring function (Meng et al., 2012; Ferreira et al., 2015). Albeit relatively fast to perform and computationally effortless when compared to other techniques like molecular dynamics, pose scoring can suffer from inaccuracies (Chaput and Mouawad, 2017). Moreover, docking neglects the role of the solvent and RNA’s plasticity, preventing the exploration of its conformational landscape (Bissaro et al., 2020; Pavan et al., 2022a).

To overcome the limitations of a docking approach, molecular dynamics simulations can be theoretically exploited to characterize RNA-ligand interactions with a more physically rigorous methodology. However, the computational effort required to spontaneously sample both binding and unbinding processes during unbiased MD simulations makes this technique incompatible with the timings of modern-day drug discovery campaigns (Bernardi et al., 2015).

Enhanced sampling algorithms are thus exploited to cut simulation times without altering the technique’s validity. These protocols rely on energetically biasing the system to increase the frequency of observation of the desired event and have been successfully applied to the study of unbinding processes in protein-ligand complexes (Do et al., 2013).

Many popular methods, including Steered Molecular Dynamics (Izrailev et al., 1999), Random Accelerated Molecular Dynamics (Lüdemann et al., 2000), and Umbrella Sampling (Torrie and Valleau, 1977), depend on the definition of so-called “collective variables” (CV), i.e., a set of descriptors that can be used to monitor the simulation and appropriately biasing the potential energy landscape. These CVs are difficult to identify since they are heavily system-dependent and rarely generally applicable (Salmaso and Moro, 2018). On the contrary, other tempering methods like Replica Exchange (Sugita and Okamoto, 1999), and Temperature Accelerated Molecular Dynamics (So/rensen and Voter, 2000), do not rely on collective variables but still require some tinkering for optimal sampling (Zamora et al., 2016).

Within the CV-free category, Thermal Titration Molecular Dynamics (TTMD) (Pavan et al., 2022c) is a recently developed method that addresses the increasing interest in the prediction of drug-target residence time, since kinetic properties such as the dissociation rate (koff) better correlate to *in vivo* ligand efficacy compared to thermodynamic properties like the equilibrium dissociation constant (Kd) (Copeland et al., 2006).

Contrary to the aforementioned techniques, the biggest perk of TTMD is its simplicity concerning simulation setup and trajectory analyses, making it more accessible to medicinal chemists without a strong modeling background. Initially developed for the comparison of protein-ligand complexes based on the persistence of their native intermolecular interaction, TTMD provides a simple and robust platform for ranking binding poses upon a defined receptor binding site (Menin et al., 2023) or classifying ligands based on their dissociation rate (Pavan et al., 2022c). Specifically, TTMD operates through a series of short classic molecular dynamics simulations performed at increasing temperatures while monitoring the persistence of native receptor-ligand interactions through an interaction fingerprints-based scoring function (Pavan et al., 2022b).

Due to the encouraging success observed in the characterization of protein-ligand complex stability with TTMD and based on the successful repurposing of various computational tools designed to work on proteins to the study of RNA targets (Mlýnský and Bussi, 2018), in the present work we aim to extend the applicability domain of the technique to the world of RNA-ligand complexes. In detail, we tried to understand if TTMD can be used as a post-docking filter to steer the identification of a native-like binding pose for small organic ligands onto RNA receptor binding sites.

## 2 Materials and methods

### 2.1 Hardware overview

Modeling tasks such as the structure preparation of RNA targets and respective ligands, docking, system setup for molecular dynamics (MD) simulations, and subsequent trajectory analysis were carried out on a Linux workstation running Ubuntu 20.04 as its operating system equipped with a 20 cores Intel Core i9-9820 × 3.3 Ghz processor. MD simulations were performed exploiting an in-house GPU cluster composed of 20 NVIDIA devices ranging from GTX1080Ti to RTX3090.

### 2.2 Structure preparation

The three-dimensional structures of the targets presented in this work were retrieved from the Protein Data Bank (PDB (Berman et al., 2000)), and successfully processed through different built-in modules of the Molecular Operating Environment (Molecular Operating Environment MOE, 2022.02, 2022). At first, each inconsistency between the primary sequence and the tertiary structure was fixed through the “Structure preparation” module. Then, the “Protonate3D″ tool was exploited for adding missing hydrogens according to the most probable tautomeric and protonation state of titratable groups at pH = 7.4. Finally, every non-RNA and non-ligand residue was removed, except for K+ ions in the G-quadruplex portion of 5BJO complex, which were retained as they play a pivotal role in the stabilization of these non-canonical structures that shape the binding site at the monomer-monomer interface (Bhattacharyya et al., 2016).

To avoid any bias in the docking calculation that may favor a crystal-like pose, the ligands have been prepared starting from their SMILES, exploiting Open Babel (O’Boyle et al., 2011) and various QUACPAC OpenEye tools (Cadence Molecular Sciences, 2023). Open Babel was used to generate two-dimensional and three-dimensional coordinates, while “Tautomers” and “Fixpka” assigned the correct protomeric and tautomeric state at pH 7.00. Afterwards “Molcharge” set the partial charges according to the MMFF94 force field. Both the RNA and ligand were stored for docking calculations.

### 2.3 Docking calculation

A self-docking calculation was conducted through the Protein-Ligand ANT System (PLANTS (Korb et al., 2006; Korb et al., 2007; Korb et al., 2009) program, one of the state-of-the-art docking tools for nucleic acids-ligand docking (Jiang et al., 2023). The binding site was defined as a sphere of radius 10.5 Å centered around the center of mass of the crystal ligand in the experimentally solved complex. The first 5 poses according to the ChemPLP scoring function were stored for refinement through the Thermal Titration Molecular Dynamics protocol.

### 2.4 System setup for MD simulations and equilibration protocol

Several packages of Visual Molecular Dynamics (VMD (Humphrey et al., 1996)) 1.9.3 and AmberTools22 (Case et al., 2005; Case, 2022) were used to prepare each RNA-ligand complex for MD simulations.

Each nucleic atom was parametrized according to ff14SB force with χ modification tuned for RNA (χ_OL3_) field (Pérez et al., 2007; Zgarbová et al., 2011; Maier et al., 2015), while ligands parameters were assigned according to General Amber Force Field (GAFF (Wang et al., 2004)). Since the Corn Aptamer (PDB ID: 5BJO) presents a modified Uracil (I5-U17), a custom preparation was exploited following Amber’s workflow to create modified residues employing the antechamber and parmcheck2 tools, assigning parameters from ff14SB and GAFF force field.

Each RNA-ligand complex was solvated within a rectangular base prism box, with a 15 Å padding between the box border and the nearest solute atom, using the TIP3P water model (Jorgensen et al., 1983).

Sodium and chlorine monovalent ions were added to neutralize the net charge of the box, reaching the physiological salt concentration of 0.154 M. Before equilibration, each system was then subjected to 500 steps of energy minimization with the conjugate-gradient method to remove clashes and bad contacts.

Following this preparation phase, a two-step equilibration process was performed. The first phase consisted of a 0.5 ns simulation in canonical ensemble (NVT) imposing a 5 kcal mol^−1^ Å^−2^ harmonic positional restraint to each RNA and ligand atom, leaving water and ions unconstrained. In the second equilibration run, a 0.5 ns simulation was conducted in the isothermal-isobaric ensemble (NPT) constraining only the ligand and the backbone atoms of RNA through the same force field constant used in the first stage. For each equilibration stage, the temperature was kept constant at the lowest value indicated in the temperature range through a Langevin thermostat (Davidchack et al., 2009), and for the second NPT simulation, the pressure was fixed at 1 atm through a Monte Carlo barostat (Faller and de Pablo, 2002).

For each MD simulation, an integration timestep of 2 fs was used. The simulations were performed exploiting the proprietary ACEMD 3.5 engine (Harvey et al., 2009), which is based on the open-source library for molecular simulations OpenMM (Eastman et al., 2017). The M-SHAKE algorithm was used to constrain the length of bonds involving hydrogen atoms, the particle-mesh Ewald (PME (Essmann et al., 1995)) method was exploited to compute electrostatic interactions using cubic spline interpolation, and finally, a 9.0 Å cutoff was used for calculating Lennard–Jones interactions.

### 2.5 Thermal titration molecular dynamics (TTMD) simulations

Thermal Titration Molecular Dynamics (TTMD (Pavan et al., 2022c; Menin et al., 2023)) is an enhanced sampling molecular dynamics approach originally developed for the estimation of protein-ligand unbinding kinetics. This method consists of a series of short MD simulations (TTMD-steps) performed at progressively increasing temperatures. The length and the temperature for each step, defining the temperature ramp for the TTMD simulation, are defined by the user based on the knowledge of the system of interest, especially concerning the conservation of the native receptor fold throughout the whole simulation. In this work, two different temperature ramps were used: the first one, defined as “standard”, is the same ramp described in the original publication (starting temperature 300 K, final temperature 450 K, temperature increase 10 K, step length 10 ns), and a second one defined as “alternative” (starting temperature 73 K, final temperature 223 K, temperature increase 10 K, step length 10 ns). An interaction fingerprint-based scoring function (Pavan et al., 2022b) is exploited to monitor the conservation of the native binding mode. Specifically, the IFP_CS_ scoring function (Pavan et al., 2022b) is used to compare each of the binding features of each trajectory frame (encoded as protein-ligand interaction fingerprints) to the last frame of the second equilibration stage. In the context of this work, two different PLIFs were employed, specifically the InteractionFingerprint function of the Open Drug Discovery Toolkit (ODDT (Wójcikowski et al., 2015)) and the one provided by the ProLIF package (Bouysset and Fiorucci, 2021), while the cosine similarity metrics was used for the comparison, as implemented in the Scikit-learn Python library. The calculated value is then multiplied by −1 to comply with the scale of most scoring functions. In the end, the resulting score can range from −1 to 0, where −1 indicates total congruence of the PLIFs (total conservation of the binding mode) and 0 indicates that all native binding features are lost.

The simulation continues until the end of the temperature ramp is reached, or an early termination criterion is reached. Specifically, at the end of each TTMD step, the average IFPcs score is calculated for the last 10% of the step: if this value is above −0.05, the simulation is stopped as the ligand lost its original binding mode. The code for running TTMD simulations is open-source and available through the MIT license at github.com/molecularmodelingsection/TTMD.

### 2.6 Trajectory analysis, MS and IFF coefficients determination

TTMD trajectories were analyzed partially through the same Python code described in the previous paragraph and partially through the SuMD-analyzer (Pavan et al., 2022a) script available at github.com/molecularmodelingsection/SuMD-analyzer. Specifically, the MDAnalysis package (Michaud-Agrawal et al., 2011; Gowers et al., 2016) was exploited to calculate the RMSD of the receptor backbone, the ligand, and a defined set of binding site residues.

The “titration timeline” plot reports the time-dependent evolution of both the ligand, binding site and receptor backbone RMSD and of the IFP_CS_ score.

The “titration profile” plots the average IFP_CS_ score for each TTMD step against the temperature at which the step was executed. In this plot, the slope of the straight line linking the first and last point of the simulation (the MS coefficient) is extracted and used as a proxy measure for the estimation of the overall complex stability. The mathematical formulation of the MS coefficient is reported in Eq. (1).

$$MS=\frac{{mean\;IFP}_{CS}^{Tend}-\left(-1\right)}{T^{end}-T^{start}}$$ (1)

MS formula.

The MS coefficient can range from zero (indicative of a tight and persistent binding/high conservation of the native binding mode) to 1 (indicative of high volatility of the native pose). For each investigated receptor-ligand complex, five independent TTMD simulations were performed, with the average MS coefficient being calculated across three different replicates, after discarding the highest and the lowest value.

Furthermore, to complement the MS coefficient, a second one defined as IFF was calculated as defined in Eq. (2).

$$IFF=\sqrt{\frac{\sum_{t}^{T}{\left({IFP}_{CS\;t}-\;\langle {IFP}_{CS}\rangle \right)}^{2}}{T}}$$ (2)

IFF formula.

The IFF coefficient is calculated as the root mean square fluctuation of the IFP_CS_ value across the simulation to the whole run mean IFP_CS_ value. As the MS coefficient only considers the initial and the final state of the simulation, neglecting even significant fingerprint fluctuations that may suggest poor complex stability, the IFF coefficient can overcome this issue, further improving the protocol accuracy.

## 3 Results

To extend the applicability domain of Thermal Titration Molecular Dynamics to the characterization of RNA-ligand complexes, six different test cases were chosen based on the chemical diversity of the ligand and structural diversity of the receptor among the experimental complexes deposited in the Protein Data Bank. Four out of the six test cases were drawn out from the work of Bissaro et al. (Bissaro et al., 2020), specifically 2LWK, 3Q50, 5BJO, and 6E1U, while 1UUD and 1UUI were added because of the challenge provided by the high conformational freedom of the P12 and P14 ligands.

Hereafter a brief overview of the six test cases.

### 3.1 Influenza A virus RNA promoter

Influenza A virus RNA promoter has a significant role in the modulation of transcription and replication of this group of viruses belonging to the Orthomyxoviridae family. Therefore, it is considered an interesting target for the development of antiviral drugs (Lee et al., 2013). Through a fragment screening approach, 6,7-dimethoxy-2-(1-piperazinyl)-4-quinazolinamine (DPQ) was identified by Varani’s group (Lee et al., 2013). This compound has a micromolar affinity for the promoter region (K_D_ of 50.5 μM), and consequently, it is a scaffold for further development of antivirals targeting the Influenza A promoter region (PDB ID: 2LWK).

### 3.2 Corn Aptamer

The Corn aptamer is an *in vitro* selected aptamer that binds a fluorophore ligand (DFHO), with a nanomolar binding affinity (K_D_ = 70 nM) (Warner et al., 2017). Thanks to its limited cytotoxicity, the Corn-DFHO could represent a valuable imaging tool. Despite being therapeutically less interesting than the other RNA structures selected for this study, its peculiar three-dimensional organization in a quasi-symmetric dimer with a non-cationic ligand buried at the monomer-monomer interface between two G-quadruplex structures stabilized by K+ ions makes it an appealing target to apply TTMD protocol, since its stability in molecular dynamics, and the low structural flexibility of the fluorophore ligand (3,5-difluoro-4-hydroxybenzylidene imidazolinone-2-oxime) led Bissaro et al. to optimal results when applying SuMD protocol to this system (Bissaro et al., 2020).

### 3.3 Pre-queuosine 1 riboswitch

PreQ1 (Pre-queosine 1, 7-aminomethyl-7-deazaguanine) is a precursor of the hypermodified guanine nucleotide Queuosine Q), a modified nucleoside that influences anticodon-codon stability in tRNAs, hence increasing translational fidelity. Prokaryotes can synthesize PreQ1 molecules from a multienzyme pathway starting from GTP. By contrast, eukaryotes do not synthesize PreQ1 *de novo* and need to retrieve queuoine from the diet (Eichhorn et al., 2014). For this reason, PreQ1 riboswitches are only found in prokaryotes, where, by binding PreQ1, they play a fundamental role in the biosynthesis and transport of this molecule. Therefore, riboswitches are an appealing target for antibacterial drug development. Two PreQ1 riboswitch complexes have been selected, the first one (PDB ID: 3Q50) is bound to a PreQ1 precursor with a nanomolar binding affinity (K_D_ = 2 nM), the second one (PDB ID6E1U) is a hybrid riboswitch aptamer bound to a synthetic compound (HMJ) that presents a sub-micromolar affinity (K_D_ = 0.5 μM). This complex has been obtained through the deletion of nucleobase A14 and the two vicinal ones, as a direct consequence, the binding site lacks important nucleotides and, congruently to the work of Bissaro et al. (Bissaro et al., 2020), before running TTMD simulations, only the ligand was retained from this complex, and the aptamer structure was retrieved from 3Q50 crystal.

### 3.4 Tar

Tat-Tar interaction modulates the transcription process in HIV-1. Therefore, targeting Tar RNA could be an appealing pathway to follow for the development of HIV-1 therapeutics. Although combination antiretroviral therapy (cART) can significantly suppress viral load, the eradication of this virus is still challenging. Moreover, chronic long-term comorbidities can arise since viral reservoirs are not eliminated by cART. In this scenario blocking HIV transcription could be beneficial (Alanazi et al., 2021). Several efforts have been made in the characterization of this target and various small-molecule complexes have been reported in the PDB. In this work, two Tar complexes have been reported, specifically selected to assay TTMD behavior with challenging flexible ligands (P12, P14) with high conformational freedom.

The investigated cases are summarized in Table 1 and their crystal structures are reported in Table 2 to showcase the structural differences of these targets.

**TABLE 1:** Summary of the six test-cases investigated in the present study.

PDB code	Ligand PDB ID	Receptor type
1UUD Davis et al. (2004)	P14	HIV-1 TAR RNA (29 bases)
1UUI Davis et al. (2004)	P12	HIV-1 TAR RNA (29 bases)
2LWK Lee et al. (2013)	0EC	influenza A virus RNA promoter (32 bases)
3Q50 Jenkins et al. (2011)	PRF	PreQ1 riboswitch aptamer (33 bases)
5BJO Warner et al. (2017)	747	Corn RNA aptamer (36 bases)
6E1U Connelly et al. (2019)	HMJ	PreQ1 riboswitch (33 bases)

**TABLE 2:** Crystal structures of the six test-cases reported in the article.

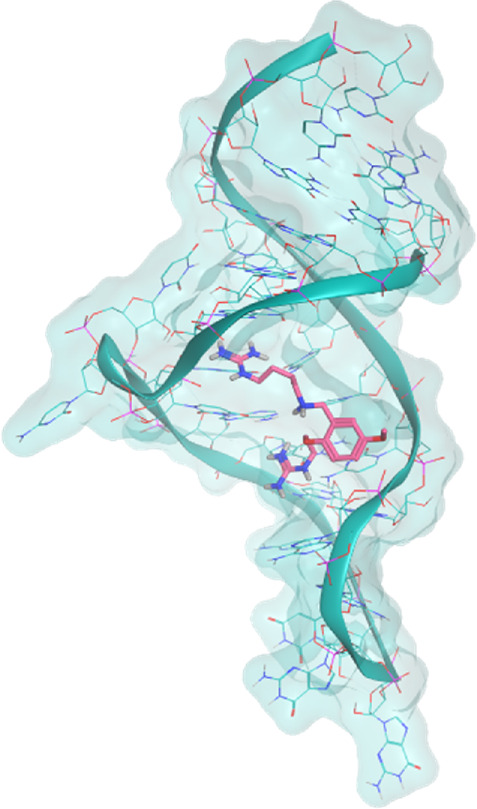	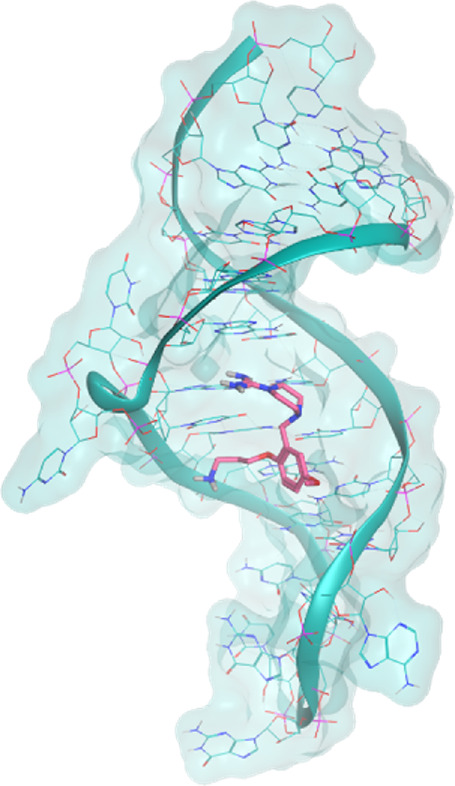	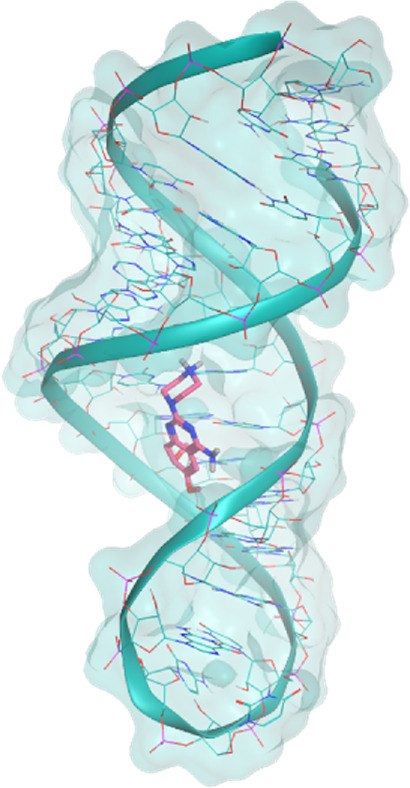
**HIV-1 TAR RNA**	**HIV-1 TAR RNA**	**Influenza A virus RNA Promoter**
**PDB ID** 1UUD	**PDB ID** 1UUI	**PDB ID** 2LWK
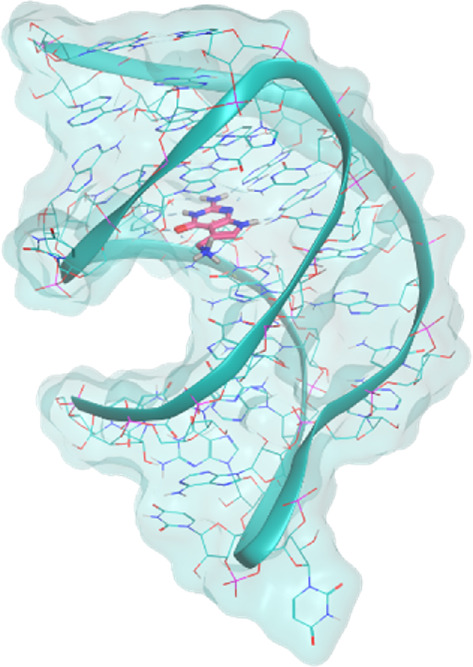	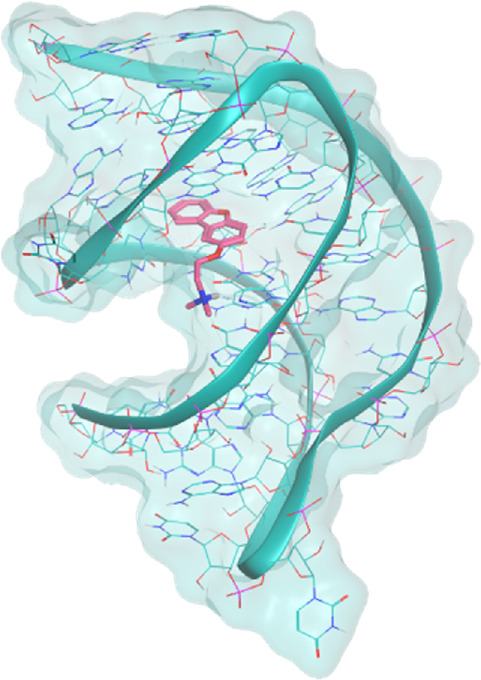	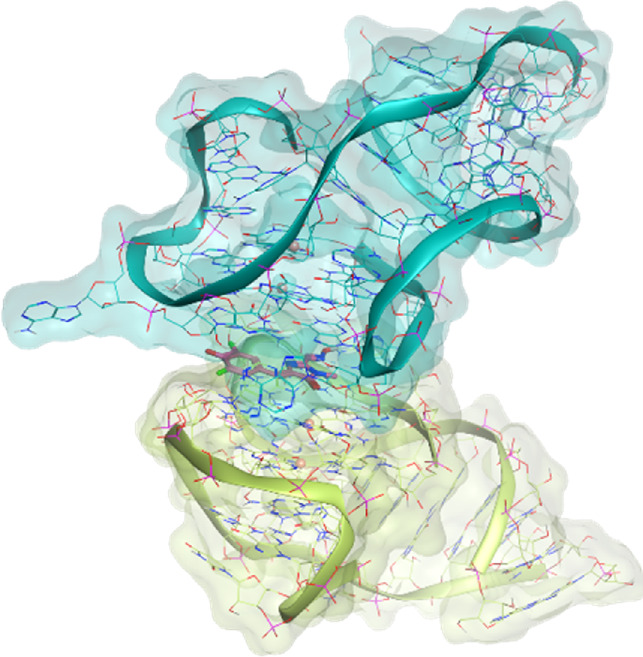
**PreQ1 Riboswitch Aptamer**	**PreQ1 Riboswitch Aptamer**	**Corn RNA Aptamer**
**PDB ID** 3Q5O	**PDB ID** 6E1U	**PDB ID** 5BJO

For each complex, at first, a self-docking experiment was conducted using the PLANTS docking program, since it has recently been reported as one of the best docking tools for RNA-ligand complexes (Jiang et al., 2023) and it is also free for academics. The results of the self-docking calculations are summarized in Tables 3–8.

**TABLE 3:** This panel encompasses all binding modes (experimental + docking poses) investigated in this work for complex deposited in the PDB with accession code 1UUD. For each pose, the ChemPLP docking score and RMSD to the experimental pose are reported.

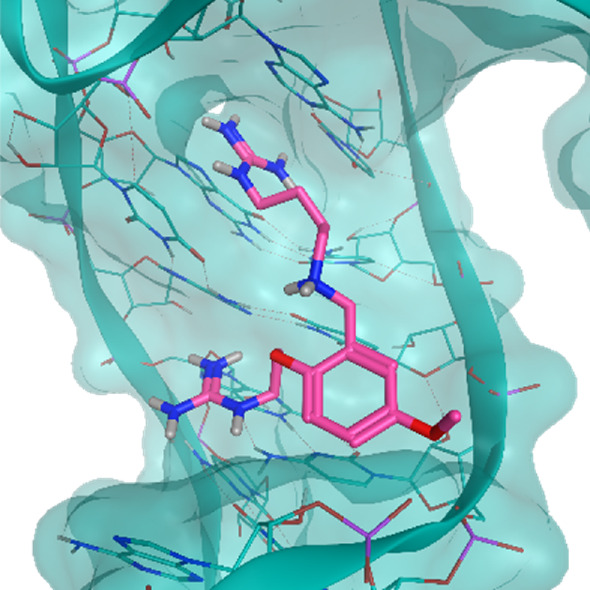	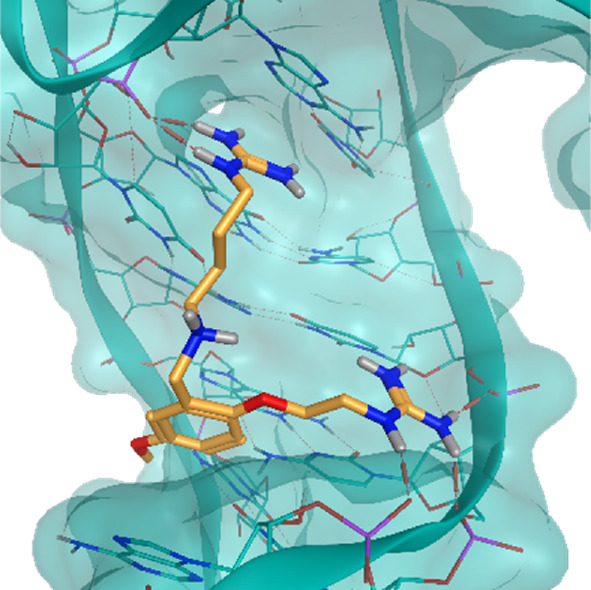	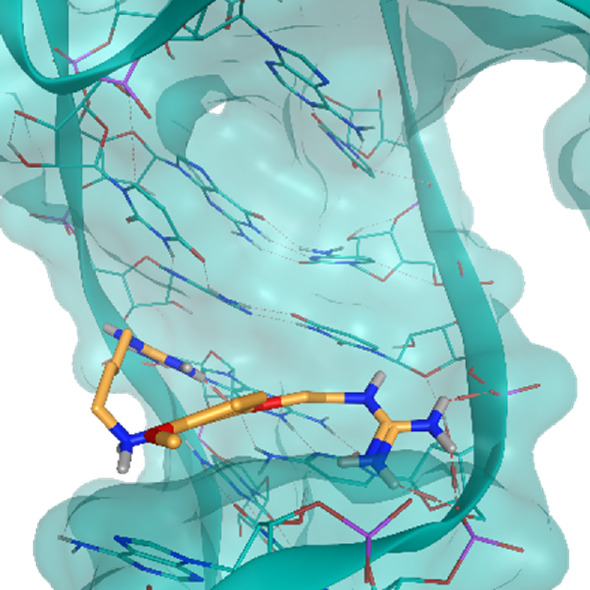
**HIV-1 TAR RNA**	**Pose 1** RMSD 5.84 Å	**Pose 2** RMSD 6.78 Å
**PDB ID** 1UUD	**ChemPLP** −86.34	**ChemPLP** −84.98
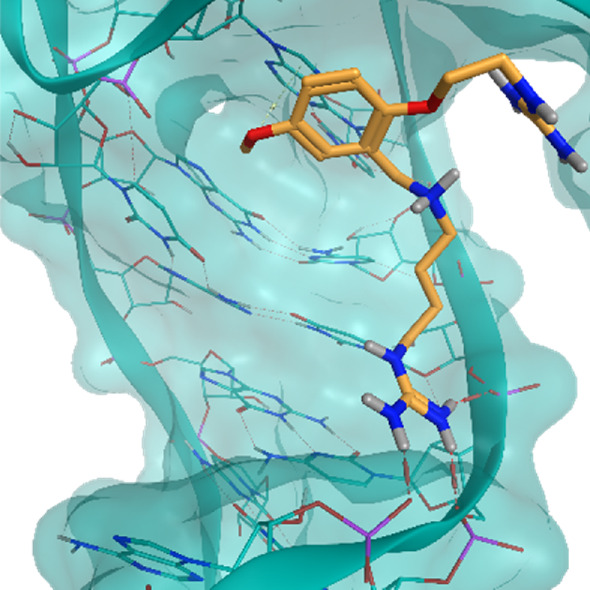	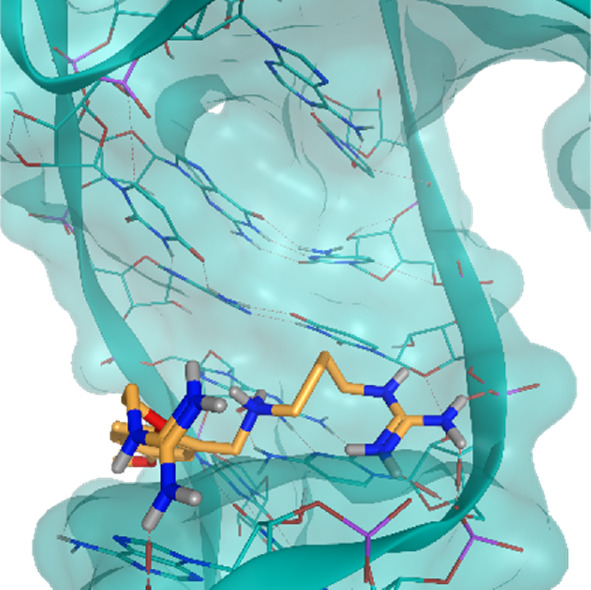	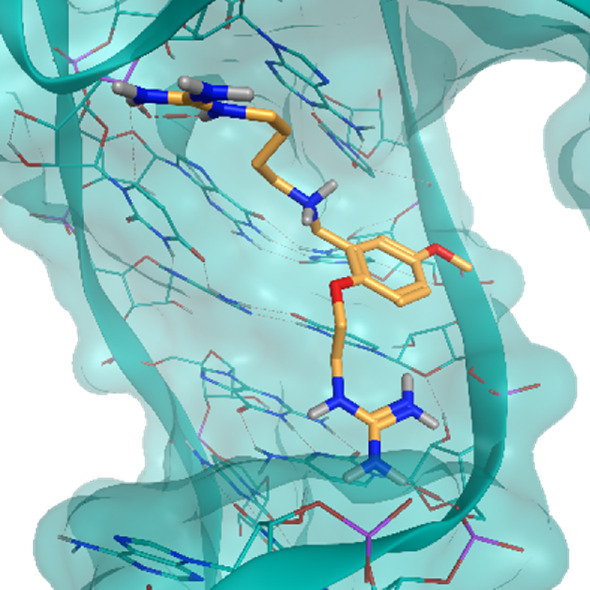
**Pose 3** RMSD 10.05 Å	**Pose 4** RMSD 8.16 Å	**Pose 5** RMSD 5.76 Å
**ChemPLP** −83.74	**ChemPLP** −83.71	**ChemPLP** −82.96

**TABLE 4:** This panel encompasses all binding modes (experimental + docking poses) investigated in this work for complex deposited in the PDB with accession code 1UUI. For each pose, the ChemPLP docking score and RMSD to the experimental pose are reported.

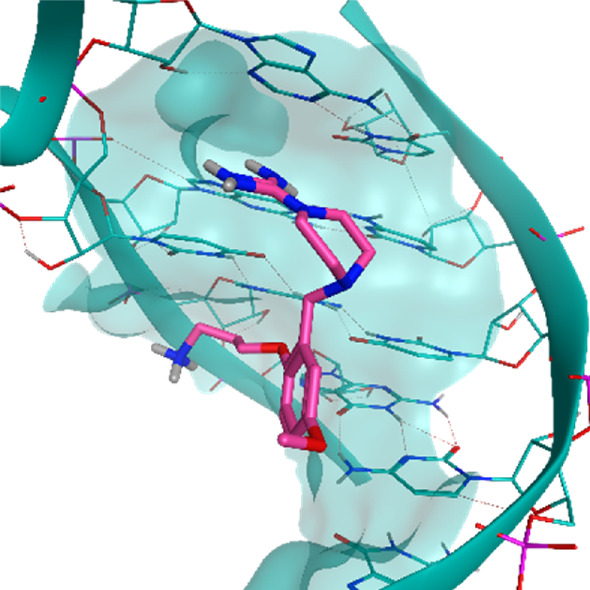	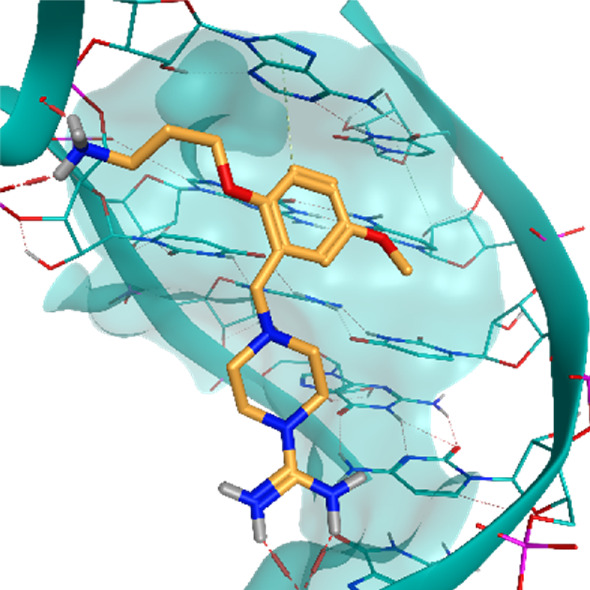	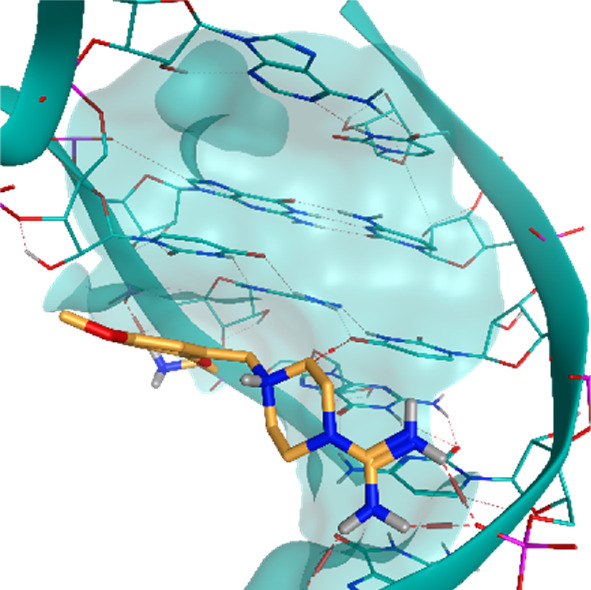
**HIV-1 TAR RNA PDB ID** 1UUI	**Pose 1** RMSD 7.05 Å **ChemPLP** −82.64	**Pose 2** RMSD 6.00 Å **ChemPLP** −79.30
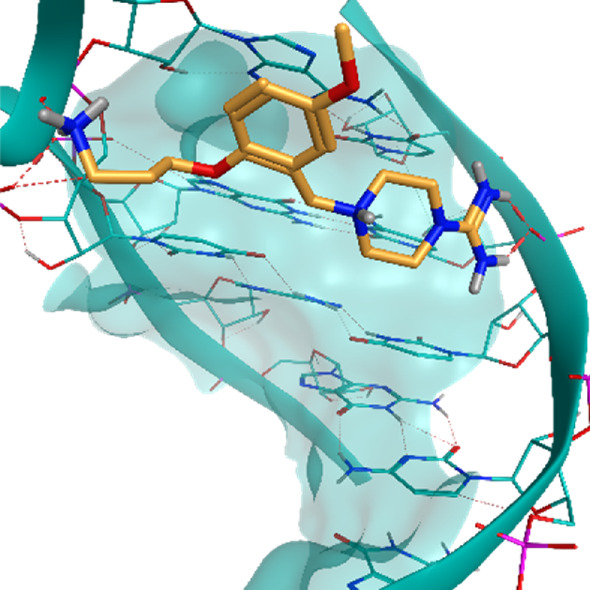	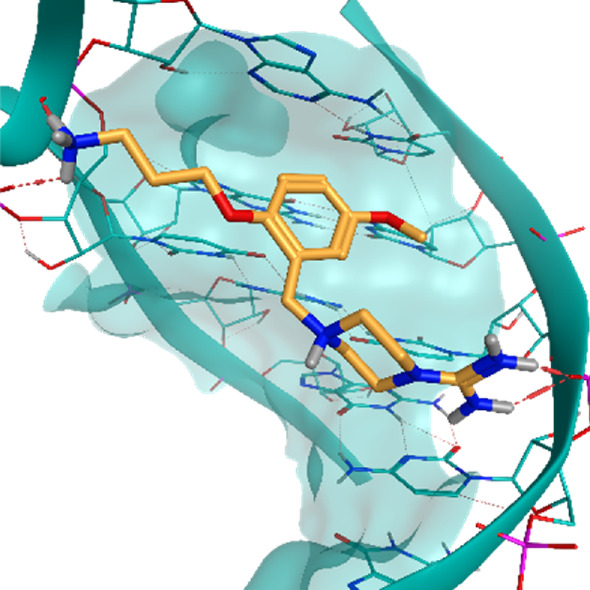	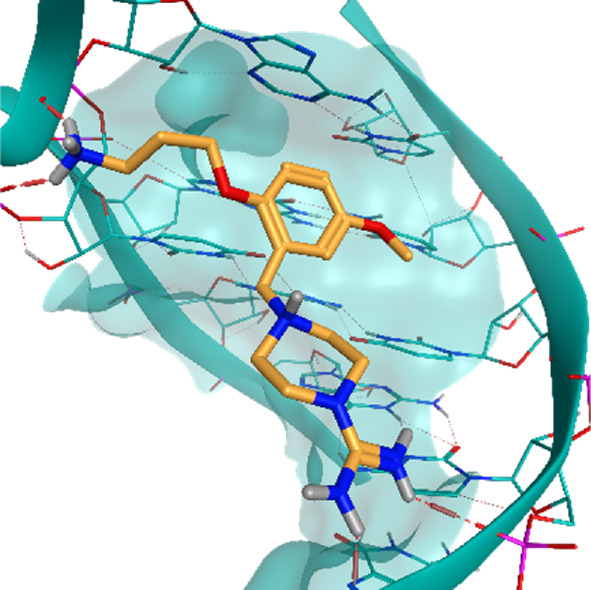
**Pose 3** RMSD 7.46 Å **ChemPLP** −79.12	**Pose 4** RMSD 6.86 Å **ChemPLP** −78.30	**Pose 5** RMSD 7.07 Å **ChemPLP** −77.83

**TABLE 5:** This panel encompasses all binding modes (experimental + docking poses) investigated in this work for complex deposited in the PDB with accession code 2LWK. For each pose, the ChemPLP docking score and RMSD to the experimental pose are reported.

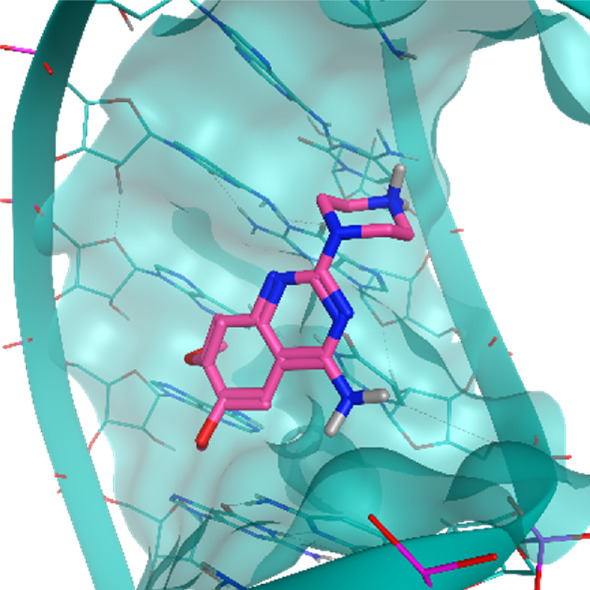	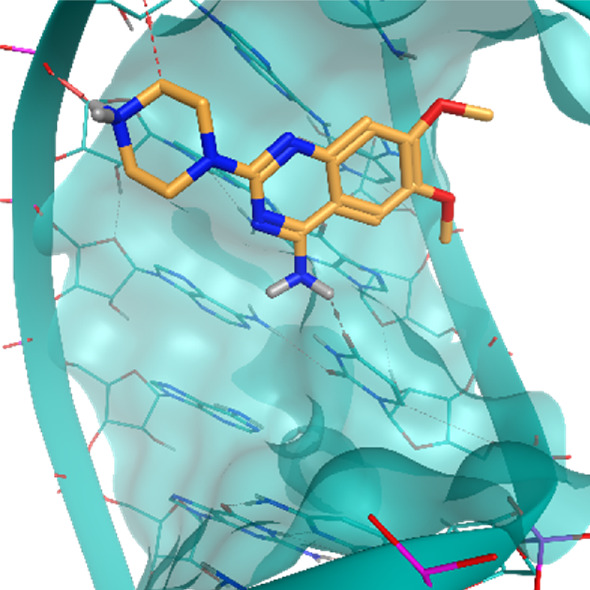	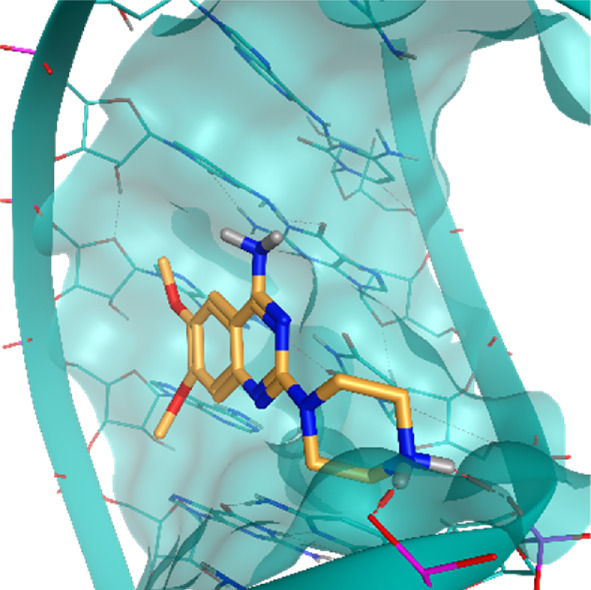
**Influenza A virus RNA Promoter PDB ID** 2LWK	**Pose 1** RMSD 7.16 Å **ChemPLP** −59.84	**Pose 2** RMSD 4.56 Å **ChemPLP** −58.85
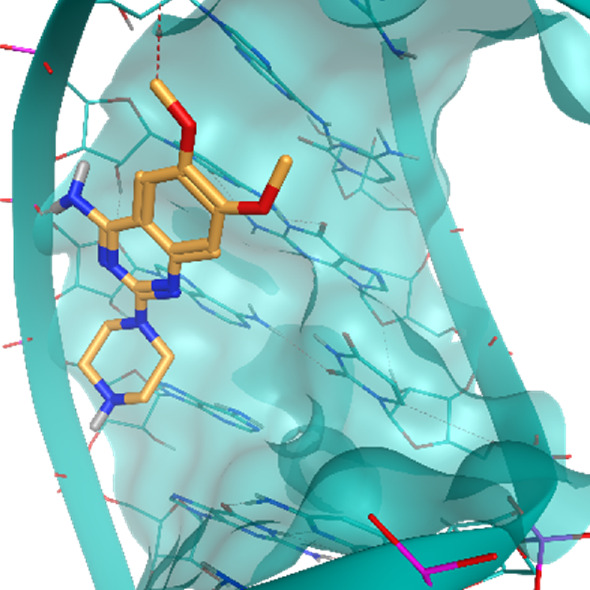	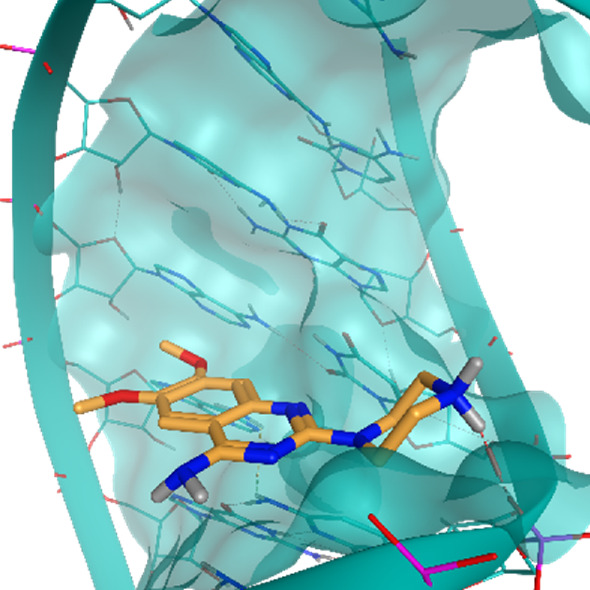	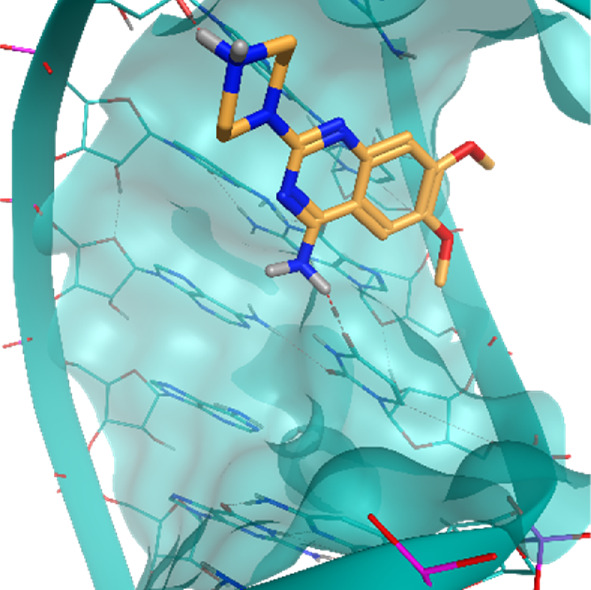
**Pose 3** RMSD 7.95 Å **ChemPLP** −57.09	**Pose 4** RMSD 4.29 Å **ChemPLP** −56.60	**Pose 5** RMSD 6.93 Å **ChemPLP** −56.37

**TABLE 6:** This panel encompasses all binding modes (experimental + docking poses) investigated in this work for complex deposited in the PDB with accession code 3Q50. For each pose, the ChemPLP docking score and RMSD to the experimental pose are reported.

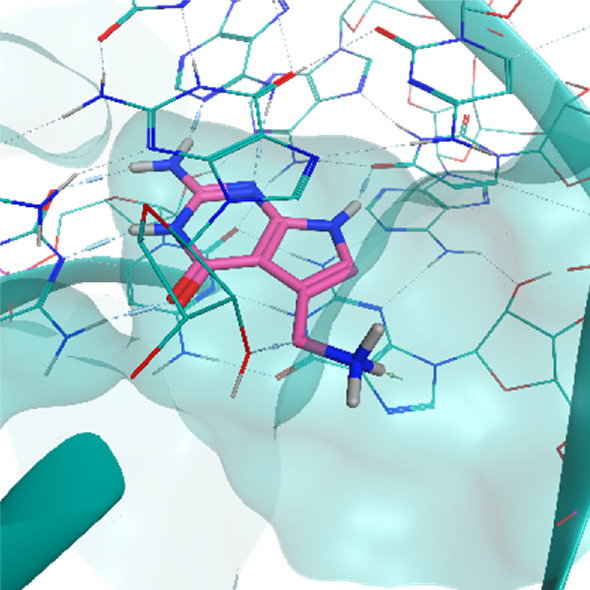	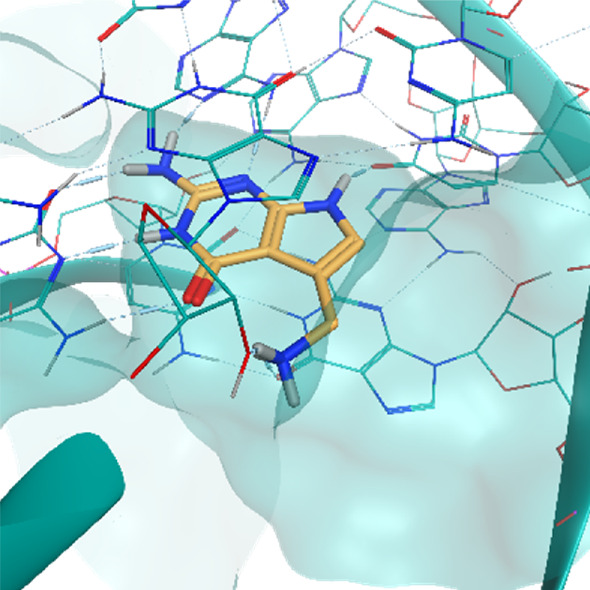	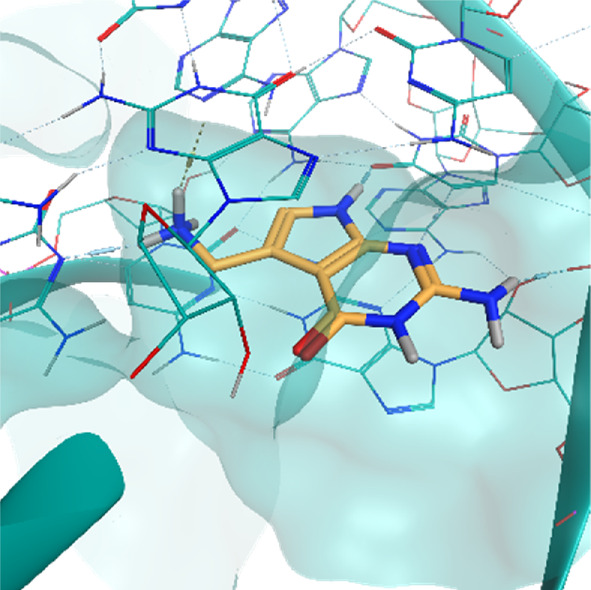
**PreQ1 Riboswitch Aptamer PDB ID** 3Q50	**Pose 1** RMSD 0.69 Å **ChemPLP** −84.96	**Pose 2** RMSD 4.58 Å **ChemPLP** −77.76
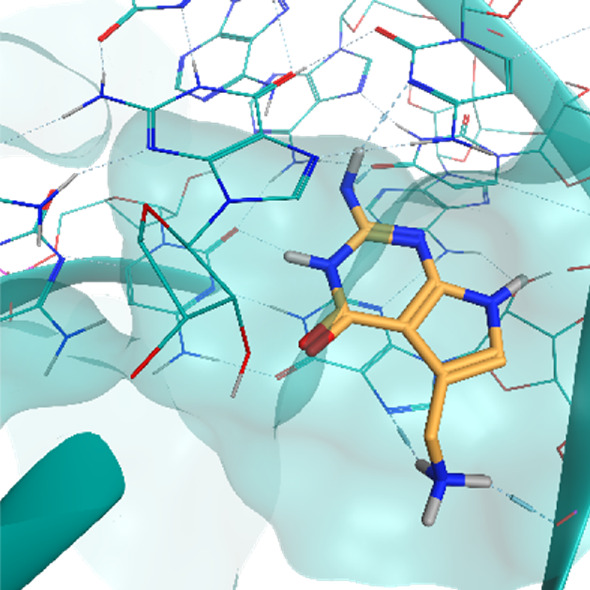	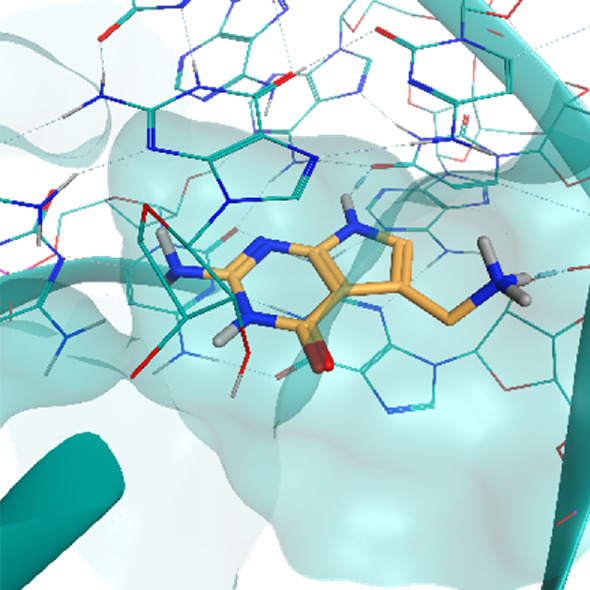	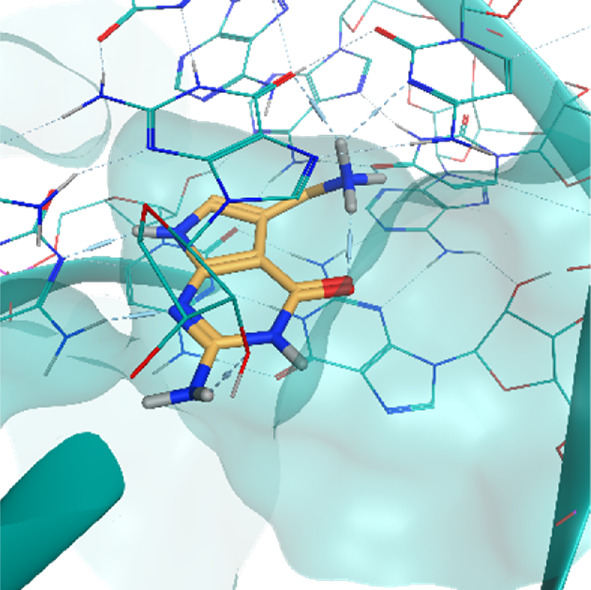
**Pose 3** RMSD 4.14 Å **ChemPLP** −73.68	**Pose 4** RMSD 2.82 Å **ChemPLP** −71.95	**Pose 5** RMSD 4.17 Å **ChemPLP** −67.95

**TABLE 7:** This panel encompasses all binding modes (experimental + docking poses) investigated in this work for complex deposited in the PDB with accession code 5BJO. For each pose, the ChemPLP docking score and RMSD to the experimental pose are reported.

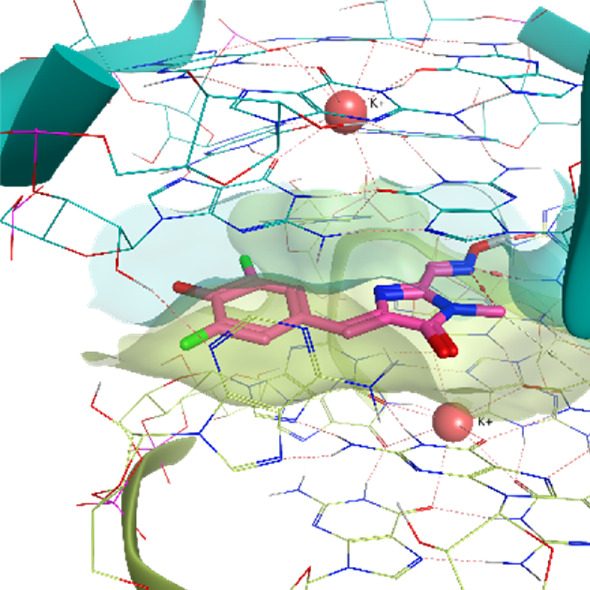	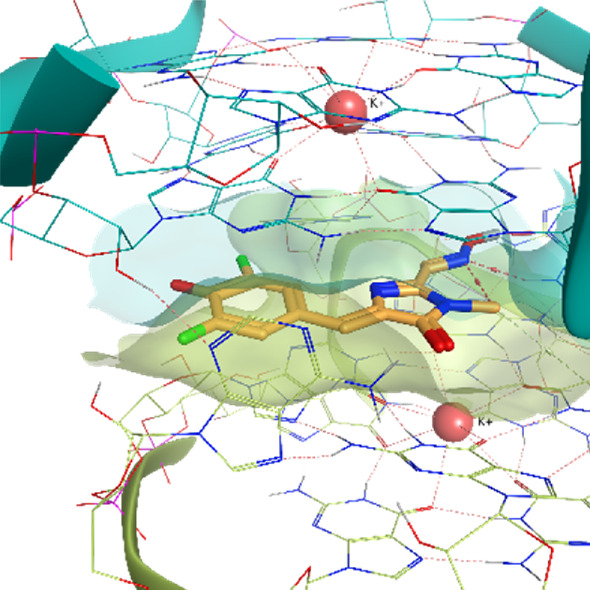	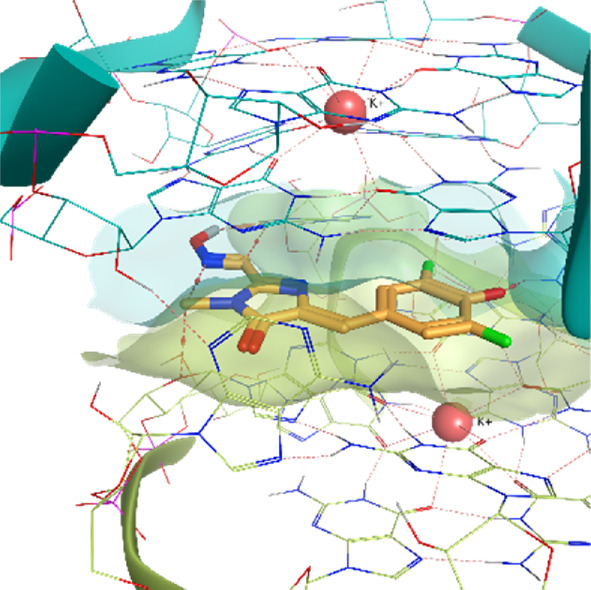
**Corn RNA Aptamer PDB ID** 5BJO	**Pose 1** RMSD 0.29 Å **ChemPLP** −107.30	**Pose 2** RMSD 6.66 Å **ChemPLP** −104.95
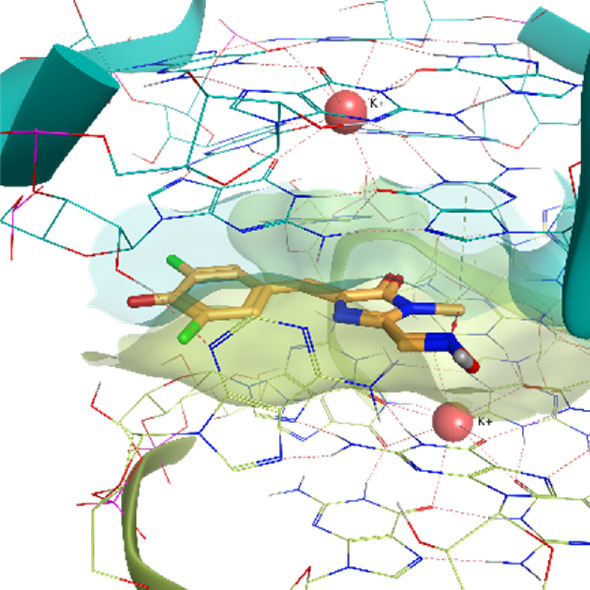	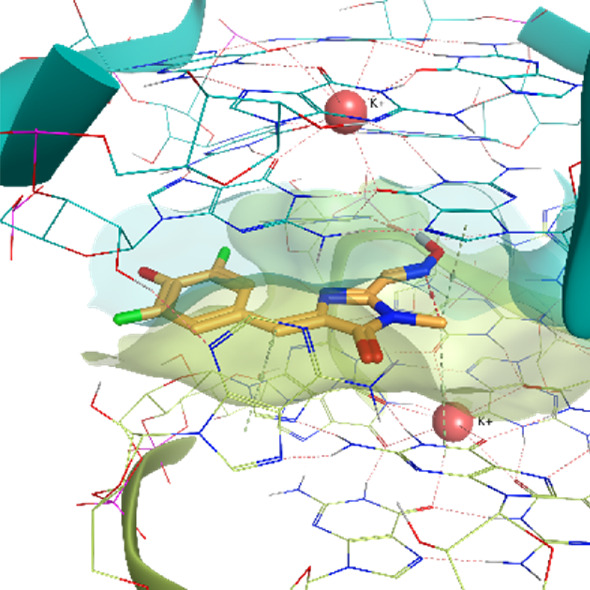	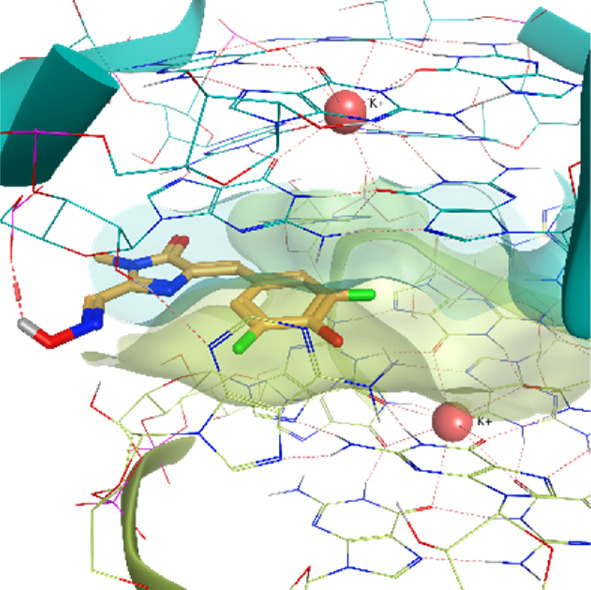
**Pose 3** RMSD 3.23 Å **ChemPLP** −85.03	**Pose 4** RMSD 1.93 Å **ChemPLP** −83.33	**Pose 5** RMSD 8.13 Å **ChemPLP** −81.20

**TABLE 8:** This panel encompasses all binding modes (experimental + docking poses) investigated in this work for complex deposited in the PDB with accession code 6E1U. For each pose, the ChemPLP docking score and RMSD to the experimental pose are reported.

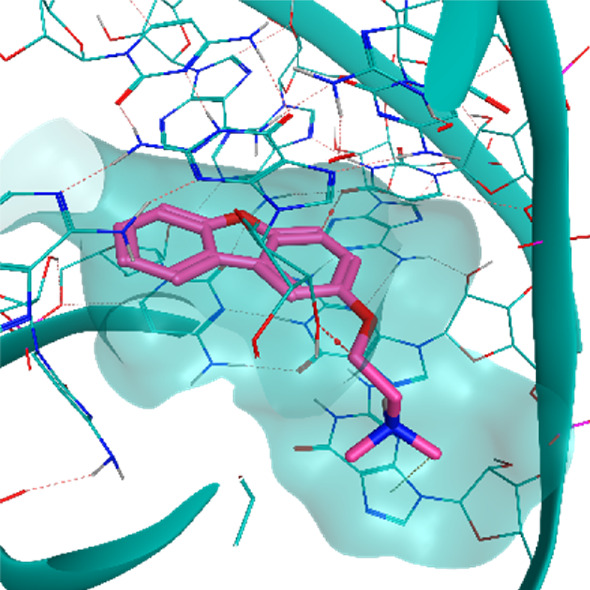	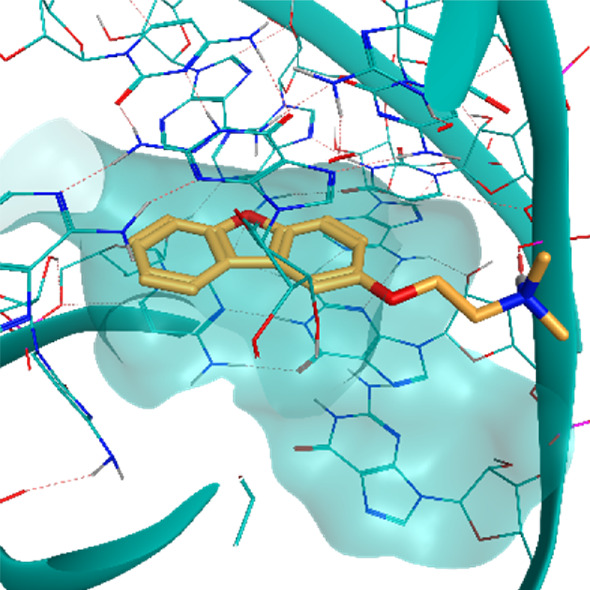	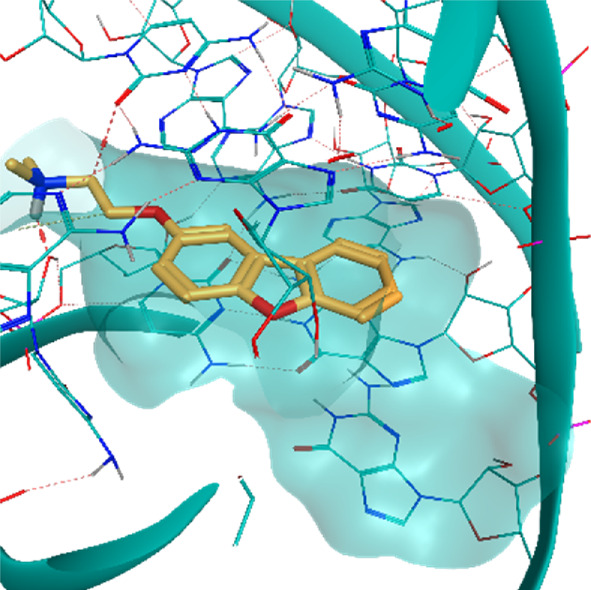
**PreQ1 Riboswitch Aptamer PDB ID** 6E1U	**Pose 1** RMSD 2.73 Å **ChemPLP** −99.21	**Pose 2** RMSD 7.99 Å **ChemPLP** −93.30
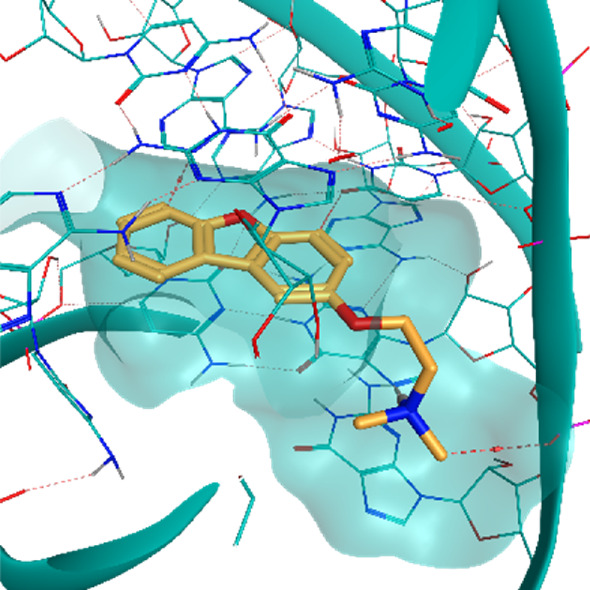	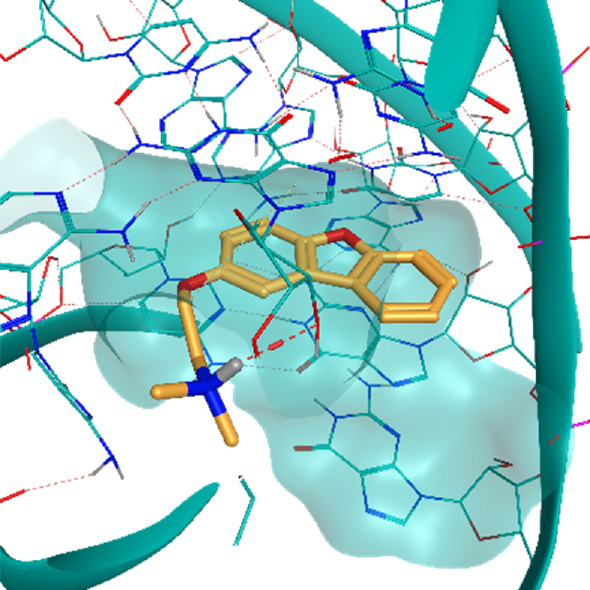	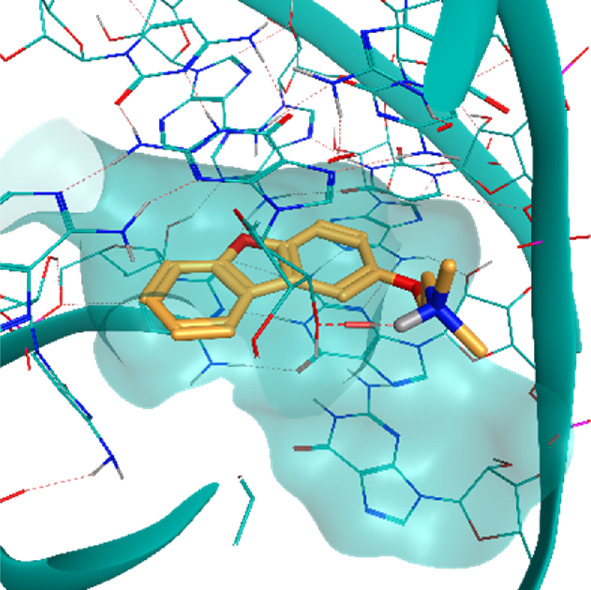
**Pose 3** RMSD 0.74 Å **ChemPLP** −92.00	**Pose 4** RMSD 5.43 Å **ChemPLP** −88.21	**Pose 5** RMSD 2.77 Å **ChemPLP** −88.19

For each complex, the top five scoring poses and the experimentally determined one were subjected to TTMD refinement using the same conditions described in the original publications (Pavan et al., 2022c; Menin et al., 2023). Furthermore, another run of TTMD calculations was conducted using an alternative temperature ramp. Two different sets of protein-ligand fingerprints were used for monitoring the binding mode’s evolution and two different metrics for the qualitative estimation of pose stability, namely, the MS and IFF coefficient. Every methodological detail is reported in the Materials and Methods section. Hereafter, each TTMD experiment is discussed separately based on execution conditions, while aggregated considerations are reported in the Discussion.

### 3.5 TTMD protocol 1: standard temperature ramp, ODDT fingerprints, MS coefficient

As a first attempt, we tried repurposing the same TTMD workflow described in the original publications (Pavan et al., 2022c; Menin et al., 2023) without any modifications. We therefore used a standard temperature ramp, setting up the starting simulation temperature at 300 K, the end temperature at 450 K, the temperature increase between each TTMD-step to 10 K, and the duration of each TTMD-step at 10 ns. Poses were then ranked according to the MS coefficient, using ODDT interaction fingerprints for monitoring the evolution of the binding mode. Results for this first round of TTMD simulations are summarized in Table 9.

**TABLE 9:** This table summarizes results for TTMD simulations carried out under the protocol 1 simulation and analysis conditions. For each investigated system, the individual MS coefficient for each TTMD replicate and the average MS value are reported. The closest value to the mean is highlighted in bold.

Model	Pose	MD1	MD2	MD3	MD4	MD5	TrimMean	TrimError
1UUD	crystal	0.00759	**0.00909**	0.01960	0.01667	0.00514	0.01112	0.00554
1UUD	1	**0.00905**	0.00628	0.01200	0.00506	0.01000	0.00844	0.00251
1UUD	2	**0.01595**	0.03264	0.00612	0.00665	0.02471	0.01577	0.01030
1UUD	3	0.00701	0.00648	0.01990	0.01661	**0.01111**	0.01157	0.00528
1UUD	4	**1.00000**	**1.00000**	**1.00000**	**1.00000**	0.09940	1.00000	0.36024
1UUD	5	0.00456	0.00612	0.00421	1.00000	1.00000	0.33689	0.48747
1UUI	crystal	**0.01000**	0.01363	**0.01000**	0.01111	0.00347	0.01037	0.00336
1UUI	1	0.01419	0.01246	**1.00000**	**1.00000**	**1.00000**	0.67140	0.48337
1UUI	2	0.01429	0.09650	0.10000	0.01664	**0.01991**	0.04435	0.03989
1UUI	3	**1.00000**	**1.00000**	**1.00000**	**1.00000**	**1.00000**	1.00000	0.00000
1UUI	4	1.00000	**0.04945**	0.03333	1.00000	0.03249	0.36093	0.47111
1UUI	5	**1.00000**	**1.00000**	0.09894	**1.00000**	0.10000	0.70000	0.44117
2LWK	crystal	1.00000	N/A	1.00000	1.00000	0.03330	1.00000	0.41859
2LWK	1	**1.00000**	**1.00000**	**1.00000**	**1.00000**	0.01906	1.00000	0.39238
2LWK	2	1.00000	1.00000	0.00833	1.00000	N/A	1.00000	0.42941
2LWK	3	0.05000	**1.00000**	**1.00000**	0.02500	**1.00000**	0.68333	0.47159
2LWK	4	1.00000	0.00598	1.00000	N/A	1.00000	1.00000	0.43042
2LWK	5	0.02000	0.09918	0.00714	1.00000	**0.03333**	0.05084	0.38534
3Q50	crystal	0.00326	**0.00237**	0.00323	0.00212	0.00168	0.00257	0.00063
3Q50	1	**0.00231**	0.00154	0.00271	0.00311	0.00216	0.00239	0.00053
3Q50	2	0.00225	0.00137	0.00210	**0.00207**	0.00198	0.00205	0.00031
3Q50	3	0.00524	**0.00480**	0.00430	0.00540	0.00233	0.00478	0.00111
3Q50	4	0.00517	**0.00404**	0.00368	0.00437	0.00353	0.00403	0.00058
3Q50	5	0.00348	0.00440	0.00314	0.00398	**0.00351**	0.00366	0.00044
5BJO	crystal	0.00318	0.00463	**0.00433**	0.00305	0.00455	0.00402	0.00069
5BJO	1	0.00273	0.00457	**0.00385**	0.00471	0.00279	0.00374	0.00085
5BJO	2	0.00391	0.00511	0.00473	0.00431	**0.00472**	0.00459	0.00041
5BJO	3	0.00368	0.00474	0.00469	**0.00444**	0.00430	0.00448	0.00038
5BJO	4	**0.00337**	0.00346	0.00415	0.00257	0.00256	0.00313	0.00060
5BJO	5	0.00371	0.00392	0.00520	0.00601	**0.00503**	0.00472	0.00085
6EIU	crystal	**0.00207**	0.00073	0.00241	0.00063	0.00653	0.00174	0.00215
6EIU	1	0.00182	0.09966	**0.02446**	0.03333	0.00064	0.01987	0.03614
6EIU	2	0.00650	0.00667	0.00382	**0.00461**	0.00390	0.00500	0.00124
6EIU	3	**0.00071**	0.00139	0.00113	0.00040	0.00055	0.00080	0.00037
6EIU	4	0.09758	0.04980	1.00000	**0.05000**	0.04929	0.06579	0.37579
6EIU	5	0.09838	1.00000	0.00250	**0.04971**	0.00427	0.05079	0.38612

As can be seen in Table 9, the standard TTMD protocol that was successfully applied to the characterization of protein-ligand complexes’ residence time cannot be readily repurposed to RNA-ligand complexes. What we noticed immediately, indeed, was that several replicates presented remarkably high MS values (1, for example), implying that the ligands lose most or even all the native interaction features in the first TTMD step. By visually inspecting the trajectory and subsequent analyses, we noticed that this was not true, as the ligands were still in place and in a conformation within the binding site that was compatible with a similar interaction pattern as the one of the reference frame. We carefully checked the fingerprint calculation step, and we noticed that ODDT interaction fingerprints cannot capture most of the interactions that exist between the ligand and the RNA receptor, especially concerning hydrogen bonds and pi stacking, which are dominant in this kind of system and captured just a fraction of the hydrophobic contacts. We tried manually modifying the cutoffs for the calculation of such interactions, to see if they were too strict, but the results did not dramatically change. We therefore concluded that it was an atom-typing issue, with ODDT not being able to rightfully classify all RNA atoms and depict the intermolecular interactions involving them. For this reason, we modified the TTMD code to use a different protein-ligand interaction fingerprint, provided by the ProLIF package (Bouysset and Fiorucci, 2021), since it supports DNA and RNA molecules as well.

### 3.6 TTMD protocol 2: standard temperature ramp, PROLIF fingerprints, MS coefficient

As a second attempt, we re-analyzed existing TTMD trajectories monitoring the evolution of the receptor-ligand interaction fingerprint through the ProLIF package instead of the ODDT one. The result of this analysis is reported in Table 10.

**TABLE 10:** This table summarizes results for TTMD simulations carried out under the protocol 2 simulation and analysis conditions. For each investigated system, the individual MS coefficient for each TTMD replicate and the average MS value are reported. The closest value to the mean is highlighted in bold.

Model	Pose	MD1	MD2	MD3	MD4	MD5	TrimMean	TrimError
1UUD	crystal	0.00553	**0.00895**	0.01250	0.01404	0.00518	0.00899	0.00358
1UUD	1	0.00833	0.00538	**0.00752**	0.00612	0.00991	0.00732	0.00160
1UUD	2	0.00596	0.03247	**0.00752**	0.00666	0.01096	0.00838	0.01002
1UUD	3	**0.00706**	0.00585	0.00392	0.01102	0.01111	0.00798	0.00285
1UUD	4	**0.01429**	0.00961	0.09604	0.01193	0.09968	0.04075	0.04213
1UUD	5	0.00470	0.00731	0.00420	**0.00694**	1.00000	0.00632	0.39769
1UUI	crystal	0.00769	0.01427	0.01000	**0.00958**	0.00336	0.00909	0.00354
1UUI	1	0.00534	0.00553	1.00000	**0.01363**	0.05000	0.02305	0.39290
1UUI	2	**0.01603**	0.00627	0.10000	0.00865	0.01954	0.01474	0.03528
1UUI	3	0.00692	0.00414	1.00000	**0.00894**	0.09760	0.03782	0.38984
1UUI	4	1.00000	**0.00709**	0.02424	0.00442	0.00602	0.01245	0.39589
1UUI	5	1.00000	0.10000	**0.03278**	0.00587	0.01250	0.04843	0.38632
2LWK	crystal	1.00000	**0.03235**	0.10000	0.00529	0.02500	0.05245	0.38506
2LWK	1	**1.00000**	**1.00000**	**1.00000**	**1.00000**	0.00833	1.00000	0.39667
2LWK	2	0.00887	0.00448	0.00833	**0.00566**	0.00533	0.00644	0.00174
2LWK	3	0.00909	**0.01588**	0.01246	0.02438	1.00000	0.01757	0.39385
2LWK	4	1.00000	0.00406	0.01111	**0.01374**	0.10000	0.04162	0.38870
2LWK	5	0.02000	0.03268	0.00694	**0.02424**	0.03307	0.02564	0.00962
3Q50	crystal	**0.00053**	0.00035	0.00045	0.00068	0.00090	0.00055	0.00019
3Q50	1	0.00060	0.00109	0.00070	**0.00067**	0.00038	0.00065	0.00023
3Q50	2	**0.00049**	0.00054	0.00038	0.00041	0.00049	0.00047	0.00006
3Q50	3	**0.00335**	0.00340	0.00342	0.00202	0.00080	0.00292	0.00105
3Q50	4	0.00154	0.00086	0.00249	**0.00220**	0.00277	0.00208	0.00069
3Q50	5	0.00191	**0.00090**	0.00115	0.00054	0.00085	0.00096	0.00046
5BJO	crystal	0.00075	0.00048	0.00053	0.00041	**0.00051**	0.00051	0.00012
5BJO	1	**0.00069**	0.00083	0.00043	0.00057	0.00524	0.00070	0.00185
5BJO	2	**0.00293**	0.00319	0.00164	0.00397	0.00211	0.00274	0.00082
5BJO	3	**0.00226**	0.00141	0.00078	0.00229	0.00509	0.00199	0.00147
5BJO	4	0.00089	0.00085	**0.00083**	0.00075	0.00083	0.00084	0.00004
5BJO	5	0.00206	0.00139	**0.00320**	0.00627	0.00596	0.00374	0.00200
6EIU	crystal	0.00114	0.00039	0.00043	**0.00050**	0.00140	0.00069	0.00042
6EIU	1	0.00035	**0.00071**	0.00077	0.00124	0.00048	0.00066	0.00030
6EIU	2	**0.00224**	0.00663	0.00054	0.00052	0.00464	0.00247	0.00239
6EIU	3	0.00034	0.00046	0.00033	**0.00037**	0.00059	0.00039	0.00010
6EIU	4	0.00185	0.00298	**0.00213**	0.00193	0.00351	0.00235	0.00065
6EIU	5	0.00172	0.00076	0.00068	**0.00119**	0.00199	0.00122	0.00052

As can be observed in Table 10, although the sensitivity of the fingerprint-based scoring function improved compared to the traditional TTMD protocol thanks to the change of the package used for the PLIF calculation, the results of the TTMD simulations are still not satisfactory. Taking aside the first three test cases, i.e., 1UUD, 1UUI, and 2LWK for which the docking itself was not reliable thus justifying the challenging nature of the task, in the other three cases the results were promising but showed a margin of improvement. Indeed, in all these three cases, the TTMD refinement would have at best excluded a couple of wrong poses from the equation but would have attributed some other wrong poses with similar scores to the experimentally determined binding mode, resulting in an inconclusive post-docking refinement procedure. On the other hand, in the case of complex 3Q50, where the closest poses to the crystal reference are pose 1 and 4, respectively, the MS coefficient would have awarded pose 1 and 2, giving them scores similar to the experimentally determined binding pose, and higher scores to the other ones. Intriguingly, the pose ranking capabilities would have been very good in the case of complexes 5BJO and 6E1U, which were the closest poses to the experimental data (1, 4 and 3 for 5BJO, and 3, 1, and 5 for 6E1U, respectively) would have been correctly distinguished by the MS coefficient from the more incorrect ones, other than being close in MS score to the crystal reference and being correctly ranked based on the RMSD to the native binding mode. Based on the visual inspection of TTMD trajectories, and on previous experience accumulated working on similar targets (Bissaro et al., 2020; Pavan et al., 2022a), we thought that a possible solution for improving the accuracy of the prediction could be to use a different temperature ramp, specifically a less aggressive one, to reduce the amplitude of the conformational movements of the RNA receptors to favorably decouple the evolution of the three-dimensional structure of the receptor from the diffusion of the ligand from the binding site.

### 3.7 TTMD protocol 3: alternative temperature ramp, PROLIF fingerprints, MS coefficient

To further improve the predictive capabilities of the method, we decided to test out an alternative, less aggressive, temperature ramp. Specifically, we set up a starting temperature of 73 K and an end temperature of 223 K, while maintaining the same temperature increase and step duration. The ramp was purposefully chosen within a temperature interval that could be considered “extreme cold” based on a pair of assumptions: first, with highly thermostable macromolecules such as protein receptors investigated in previous TTMD works, working within a temperature interval that is way outside the realistic representation of the system did not impair the ability to extract meaningful data out of the simulation (i.e., a qualitative estimation of the residence time), second lowering the temperature of the simulated system will reduce the atomic velocities, thus resulting in a decreased flexibility of the RNA receptor. Although temperatures in Molecular Dynamics are not directly equivalent to the real-world ones, a similar concept of extracting meaningful data at non-physiological temperatures is commonly applied in Molecular Biology, for example, while determining crystallographic structures with X-ray spectroscopy and cryo-EM techniques. The results of this set of TTMD simulations are encompassed in Table 11.

**TABLE 11:** This table summarizes results for TTMD simulations carried out under the protocol 3 simulation and analysis conditions. For each investigated system, the individual MS coefficient for each TTMD replicate and the average MS value are reported. The closest value to the mean is highlighted in bold.

Model	Pose	MD1	MD2	MD3	MD4	MD5	TrimMean	TrimError
1UUD	crystal	**0.00442**	0.00314	0.00493	0.00436	0.00494	0.00457	0.00065
1UUD	1	0.00476	0.00090	0.00089	**0.00125**	0.00353	0.00189	0.00159
1UUD	2	0.00461	0.00397	0.00324	0.00279	**0.00336**	0.00352	0.00063
1UUD	3	0.00534	0.00159	0.00318	0.00144	**0.00159**	0.00212	0.00150
1UUD	4	0.00068	0.00221	0.00686	**0.00239**	0.00280	0.00247	0.00206
1UUD	5	0.00449	0.00189	0.00282	0.00440	**0.00299**	0.00340	0.00099
1UUI	crystal	0.00402	0.00353	0.00321	0.00191	**0.00329**	0.00335	0.00070
1UUI	1	**0.00192**	0.00175	0.00167	0.00200	0.00198	0.00188	0.00013
1UUI	2	0.00054	**0.00073**	0.00058	0.00109	0.00555	0.00080	0.00193
1UUI	3	0.00282	0.00349	0.00135	0.00323	**0.00302**	0.00302	0.00075
1UUI	4	0.00355	0.00157	0.00302	**0.00255**	0.00230	0.00262	0.00067
1UUI	5	0.00205	0.00437	0.00182	0.00308	**0.00266**	0.00259	0.00090
2LWK	crystal	**0.00334**	0.00336	0.00327	0.00371	0.00279	0.00332	0.00029
2LWK	1	0.00448	**0.00452**	0.00489	0.00118	0.00500	0.00463	0.00143
2LWK	2	0.00203	0.00170	0.00506	0.00349	**0.00234**	0.00262	0.00123
2LWK	3	0.00042	0.00087	0.00437	0.00571	**0.00273**	0.00266	0.00202
2LWK	4	0.00133	0.00142	0.00089	**0.00112**	0.00091	0.00112	0.00021
2LWK	5	**0.00381**	0.00343	0.00445	0.00199	0.00517	0.00390	0.00107
3Q50	crystal	0.00072	**0.00026**	0.00015	0.00060	0.00024	0.00037	0.00022
3Q50	1	**0.00016**	0.00017	0.00065	0.00016	0.00012	0.00016	0.00020
3Q50	2	0.00024	0.00041	0.00045	**0.00033**	0.00023	0.00032	0.00009
3Q50	3	0.00029	0.00037	**0.00041**	0.00046	0.00051	0.00041	0.00008
3Q50	4	0.00131	0.00143	**0.00139**	0.00164	0.00127	0.00138	0.00013
3Q50	5	0.00037	**0.00049**	0.00060	0.00055	0.00043	0.00049	0.00008
5BJO	crystal	0.00028	**0.00020**	0.00015	0.00021	0.00017	0.00019	0.00004
5BJO	1	**0.00019**	0.00021	0.00022	0.00016	0.00017	0.00019	0.00002
5BJO	2	0.00150	0.00290	0.00221	**0.00175**	0.00156	0.00184	0.00052
5BJO	3	0.00061	0.00038	**0.00047**	0.00051	**0.00047**	0.00049	0.00007
5BJO	4	0.00099	0.00099	0.00044	**0.00071**	0.00067	0.00079	0.00021
5BJO	5	0.00095	**0.00141**	0.00037	0.00161	0.00358	0.00133	0.00108
6EIU	crystal	0.00037	0.00032	0.00022	0.00016	**0.00027**	0.00027	0.00008
6EIU	1	0.00020	0.00055	**0.00028**	0.00023	0.00032	0.00028	0.00012
6EIU	2	0.00019	0.00053	0.00032	0.00042	**0.00039**	0.00038	0.00011
6EIU	3	**0.00016**	**0.00016**	0.00224	0.00015	0.00039	0.00024	0.00082
6EIU	4	0.00103	0.00086	0.00175	**0.00167**	0.00191	0.00149	0.00042
6EIU	5	0.00059	0.00049	0.00076	0.00044	**0.00054**	0.00054	0.00011

As can be deducted from Table 11, the use of an alternative temperature ramp that operates at lower temperature values did not necessarily improve the quality of the TTMD post-docking refinement. First, the quality of the prediction decreased in those cases (1UUD, 1UUI, and 2LWK) where docking was not able to generate a good docking pose: indeed, while protocol 2 was at least able to assign the lowest scores to a pool of poses that included the experimentally determined ones, here it can be noticed how the native binding mode is always among the ones with the worst scores in term of MS coefficient. Speaking of the other three cases, instead, where protocol 2 performed quite decently, here the pose ranking capabilities of TTMD stay identical, with the only difference being that the MS score difference between various poses is decreased, flattening the difference in score between native-like poses and wrong ones. Looking at the fingerprint profile, though, we noticed how lowering the temperature ranges not only reduced the amplitude of conformational shifts in the nucleic receptor but also decreased the tendency of the ligand to diffuse from the binding site, thus making the protocol less efficient. Practically speaking, although the “titration timeline” profile clearly showed a difference in the amplitude of the changes in the fingerprint-based score throughout the TTMD simulation, the difference between the end and starting point of the simulation was not so marked. This observation led us to conclude that, maybe, the MS coefficient in its original formulation could not be the most sensitive metric to capture the subtle rearrangements of the ligand within the binding site and the conservation of the binding features, thus inducing us to introduce a different metric defined as IFF coefficient (see Materials and Methods for a detailed explanation on how the IFF coefficient is computed).

### 3.8 TTMD protocol 4: alternative temperature ramp, PROLIF fingerprints, IFF coefficient

To test if an alternative metric to the MS coefficient would be able to improve the sensitivity of the TTMD protocol using an alternative temperature ramp that operates at sub-freezing temperature, we re-analyzed those trajectories determining the IFF coefficient. The results are summarized in Table 12.

**TABLE 12:** This table summarizes results for TTMD simulations carried out under the protocol 4 simulation and analysis conditions. For each investigated system, the individual IFF coefficient for each TTMD replicate and the average IFF value are reported. The closest value to the mean is highlighted in bold.

Model	Pose	MD1	MD2	MD3	MD4	MD5	TrimMean	TrimError
1UUD	crystal	0.08170	0.10090	0.13150	0.09300	**0.10040**	0.09810	0.01653
1UUD	1	0.06440	0.02760	**0.04400**	0.04900	0.04240	0.04513	0.01185
1UUD	2	0.20300	0.13680	**0.14390**	0.08850	0.15780	0.14617	0.03683
1UUD	3	0.12670	**0.07270**	0.09660	0.05810	0.05610	0.07580	0.02661
1UUD	4	0.09040	0.16830	0.20920	0.10370	**0.15340**	0.14180	0.04341
1UUD	5	0.22860	0.07600	0.11650	0.25030	**0.12610**	0.15707	0.06776
1UUI	crystal	0.14520	**0.13290**	0.10620	0.18700	0.10570	0.12810	0.03000
1UUI	1	0.07680	0.07460	0.06880	**0.07180**	0.06380	0.07173	0.00456
1UUI	2	0.04520	0.03630	0.08390	**0.05670**	0.26680	0.06193	0.08601
1UUI	3	0.12960	**0.10450**	0.09530	0.09370	0.15250	0.10980	0.02268
1UUI	4	0.12010	0.07550	0.06420	**0.11970**	0.12710	0.10510	0.02608
1UUI	5	**0.10760**	0.11910	0.08610	0.10380	0.11110	0.10750	0.01095
2LWK	crystal	**0.10880**	0.08440	0.14570	0.10430	0.23220	0.11960	0.05244
2LWK	1	0.20760	**0.17670**	0.16880	0.06800	0.24650	0.18437	0.05942
2LWK	2	0.09790	0.09620	0.21300	0.16450	**0.10550**	0.12263	0.04627
2LWK	3	0.03890	0.06550	0.24620	0.27600	**0.13130**	0.14767	0.09482
2LWK	4	**0.07100**	0.05820	0.08010	0.07680	0.06400	0.07060	0.00806
2LWK	5	0.19820	0.16620	**0.19210**	0.11030	0.23790	0.18550	0.04213
3Q50	crystal	0.03240	0.01460	0.01350	0.02740	**0.01560**	0.01920	0.00771
3Q50	1	0.01830	**0.01940**	0.03700	0.01800	0.02800	0.02190	0.00741
3Q50	2	0.01190	0.02160	**0.01500**	0.01570	0.01470	0.01513	0.00318
3Q50	3	0.01540	**0.02380**	0.02470	0.01890	0.02420	0.02230	0.00365
3Q50	4	0.03980	0.06500	0.06080	0.07390	**0.06200**	0.06260	0.01123
3Q50	5	0.03190	**0.02640**	0.03390	0.02570	0.02420	0.02800	0.00378
5BJO	crystal	0.01500	0.01070	**0.01290**	0.01690	0.01200	0.01330	0.00220
5BJO	1	0.01250	0.01070	0.01530	**0.01210**	0.01180	0.01213	0.00153
5BJO	2	0.05420	0.08590	**0.06550**	0.08580	0.05420	0.06850	0.01427
5BJO	3	0.02340	0.01610	0.02170	**0.02030**	0.01890	0.02030	0.00249
5BJO	4	**0.03760**	0.03790	0.01630	0.04140	0.02980	0.03510	0.00899
5BJO	5	**0.08020**	0.08120	0.05140	0.07380	0.16150	0.07840	0.03752
6EIU	crystal	0.02510	0.02530	**0.02400**	0.01210	0.01150	0.02040	0.00639
6EIU	1	0.02380	**0.01820**	0.01500	0.03590	0.01400	0.01900	0.00802
6EIU	2	**0.02140**	0.03310	0.01610	0.02880	0.02040	0.02353	0.00613
6EIU	3	0.00740	**0.01510**	0.11340	0.00900	0.01950	0.01453	0.04049
6EIU	4	0.05210	0.04850	0.08180	0.12690	**0.05890**	0.06427	0.02904
6EIU	5	0.02720	0.02240	0.03100	0.03700	**0.02890**	0.02903	0.00478

By observing Table 12, it can be seen that even the new metric is not able to improve the results of the TTMD refinement performed at much lower temperatures. Specifically, the performance of the method remains like what could be observed with the old-fashioned MS coefficient. To further prove the point, we re-analyzed those trajectories performed with the standard temperature ramp (see Supplementary Table S1) and we found a great level of coherence between the MS and IFF coefficient regarding their ability to rank poses based on the persistence of native binding features throughout the simulation.

## 4 Discussion

Molecular docking represents the state-of-the-art technique for the prediction of protein-ligand complexes, thanks to the good compromise between accuracy and rapidity of execution (Pavan and Moro, 2023). Although routinely used in various structure-based drug discovery campaigns, molecular docking has been specifically designed and optimized with protein-ligand receptor in mind, due to pharmaceutical relevance and availability of experimentally determined structures to use as a benchmark for method development (Salmaso and Moro, 2018). Despite the growing interest of medicinal chemists in targeting RNA macromolecules for therapeutic and diagnostic purposes in the last decade, the field of rational design of RNA-targeting ligands is still relatively unexplored, due to some intrinsic challenges portrayed by these molecular entities, such as their structural plasticity which makes it difficult to obtain their experimentally determined three-dimensional structure, a pivotal requirement for modern-days drug discovery campaigns (Bissaro et al., 2020; Pavan et al., 2022a).

Despite all these challenges, recent works have demonstrated how, despite some intrinsic limitations, routinely used molecular docking programs such as PLANTS can be quite efficiently used for investigating RNA-ligand complexes as well (Jiang et al., 2023). Furthermore, we previously showcased how Supervised Molecular Dynamics (SuMD) simulations can be successfully utilized to flank molecular docking in investigating the recognition process of complexes involving RNA molecules, both as ligands (Pavan et al., 2022a) and as receptors (Bissaro et al., 2020), retrieving useful information about the whole binding process beyond the final bound state. Critically, one of the major problems of docking in all its iterations, both classic and dynamic, is finding a good scoring metric that can discriminate native-like poses from the wrong ones (Chaput and Mouawad, 2017). The peculiarities of RNA molecules, such as the distinctive surface charge distribution, exacerbate the limitations of classical scoring functions, which have been developed specifically for protein binding sites. For all these reasons, introducing new and improved ways of investigating RNA-ligand complexes is a very pressing issue.

In the present article, we presented the first application of Thermal Titration Molecular Dynamics (TTMD), a recently developed MD-based protocol for the qualitative estimation of protein-ligand unbinding kinetics, to the characterization of RNA-ligand complexes. We investigated six different pharmaceutically relevant test cases of different molecular complexity, both on the ligand and on the receptor side. We performed two rounds of simulations, one with the standard temperature ramp already described in the original publications (Pavan et al., 2022c; Menin et al., 2023), and one with an alternative ramp operating at sub-freezing temperatures, to investigate the effect of the temperature ramp on the accuracy of prediction. We modified the original code to use the ProLIF package (Bouysset and Fiorucci, 2021) for the calculation of RNA-ligand interaction fingerprint instead of the ODDT one (Wójcikowski et al., 2015), since they exhibit a better description of these systems. We also explored a different metric, the IFF coefficient, to use alongside the MS coefficient for monitoring the conservation of the native binding determinants and calculating a proxy measure for the receptor-ligand residence time.

By analyzing the whole set of simulations and analysis performed upon them, it seems clear that TTMD can be a helpful tool to refine docking results in those cases where the geometry of the binding site is well defined and maintained throughout the simulation, in a similar way to what was already observed for protein-ligand systems. 5BJO simulations are proof of this concept as the binding site remains extremely stable throughout the simulation (Video V1, Supplementary Table S1, Supplementary Table S2). In those cases where the perturbation of the receptor conformation and or the receptor fold overcomes the tendency of the ligand to diffuse from the binding site, the method accuracy drops significantly, not improving docking results and even invalidating successful ones.

Concerning this aspect, the choice of the temperature ramp is pivotal for the success of the TTMD rescoring process. Despite the high flexibility of RNA molecules, especially at high simulation temperatures, the standard temperature ramp still outperformed the alternative, sub-freezing one. A plausible explanation for this evidence is that the electrostatic component dominates the protein-ligand interaction energy, making it very unlikely for the ligand to spontaneously detach and unbind without any external aid in the shape of a temperature increase. Regardless of the choice of the metric used for estimating the pose residence time (the MS or the IFF coefficient), the ranking provided by the standard ramp was more useful than the one provided by the alternative one, due to the flattening of the score differences between good and bad poses at lower temperatures. Despite this, the exploration of the alternative ramp allowed us to determine that simulating in those conditions will not result in discarding a good pose, at least for those cases where TTMD (and docking too) perform well. The possibility to tune the temperature interval with a certain degree of freedom based on the knowledge of the system makes it quite appealing, since in a prospective use case one could simply start with a titration in the low-temperature range, evaluate the separation in score between different poses, and eventually re-execute the titration at higher temperatures based on the results provided by the first round of simulations.

Another useful expediency that the user could implement would be to carry out a TTMD “dry run” on just the RNA molecule itself, a functionality that has been introduced purposefully in the latest version of the TTMD code used for this article, and evaluate its behavior in terms of structural plasticity, especially at the binding site level, before running the titration on systems including the ligand as well. It is important to stress that probably exists an intermediate ramp between the two that were utilized in the present work that might be a better compromise and provide slightly better results, but at the same time, we firmly believe that to be adopted by the largest number of user possible a method has to be easy to implement and setup, without spending too much time on optimizing parameters for its execution. For this reason, we wanted to showcase that the method can perform reasonably well almost regardless of the choice of the ramp, although the original ramp that has already proven to be successful in the case of protein-ligand complexes is still the best one in terms of accuracy. Finally, it is worth mentioning that working with macromolecules that have an intrinsic conformational flexibility such as RNA strongly limits the applicability of any given MD technique in a high-throughput fashion, due to its limited sampling capabilities, making docking still the most efficient choice for investigating these systems. On the other hand, the ever-increasing computational power available for MD simulations will make and more appealing the use of pipelines like TTMD in the future, thus justifying the interest in the further development of this and other MD-based methods for the investigation of systems involving nucleic acids (Sponer et al., 2018).

## Data Availability

The datasets presented in this study can be found in online repositories. The names of the repository/repositories and accession number(s) can be found in the article/Supplementary Material.
