# Supplementary material for: Thermal titration molecular dynamics (TTMD): shedding light on the stability of RNA-small molecule complexes
Source: Front Mol Biosci. 2023 Nov 13;10:1294543. doi: 10.3389/fmolb.2023.1294543 (PMC10679717; doi:10.3389/fmolb.2023.1294543)
Supplement: Supplementary file 1 [file DataSheet1.pdf]

Thermal Titration Molecular Dynamics (TTMD): Shedding Light on the Stability of RNA-Small Molecule Complexes

Andrea Dodaro[1], Matteo Pavan[1], Silvia Menin[1], Veronica Salmaso[1], Mattia Sturlese[1], Stefano Moro[1,*]

[1] Molecular Modeling Section (MMS), Department of Pharmaceutical and Pharmacological Sciences, University of Padova, via Marzolo 5, 35131 Padova, Italy

Supplementary Material

Model	Pose	MD1	MD2	MD3	MD4	MD5	TrimMean	TrimError
1UUD	crystal	0.13430	0.14460	0.10390	0.13620	0.13200	0.13417	0.01382
1UUD	1	0.15820	0.15230	0.29990	0.22580	0.24560	0.20987	0.05551
1UUD	2	0.14130	0.24850	0.17130	0.13280	0.16370	0.15877	0.04099
1UUD	3	0.19520	0.17360	0.15170	0.21200	0.17550	0.18143	0.02052
1UUD	4	0.19660	0.17100	0.24000	0.20140	0.18720	0.19507	0.02287
1UUD	5	0.10070	0.17550	0.15340	0.09350	0.17790	0.14320	0.03628
1UUI	crystal	0.17180	0.19140	0.18290	0.21790	0.16820	0.18203	0.01774
1UUI	1	0.25300	0.26420	0.26230	0.14290	0.19560	0.23697	0.04755
1UUI	2	0.30900	0.16960	0.31740	0.32330	0.33500	0.31657	0.06122
1UUI	3	0.20890	0.15700	0.11320	0.11990	0.20830	0.16173	0.04128
1UUI	4	0.18580	0.16900	0.21920	0.17000	0.13610	0.17493	0.02698
1UUI	5	0.13280	0.12780	0.18940	0.13130	0.12770	0.13063	0.02388
2LWK	crystal	0.10700	0.18270	0.19120	0.11020	0.24570	0.16137	0.05263
2LWK	1	0.34640	0.18240	0.24650	0.30850	0.13490	0.24580	0.07791
2LWK	2	0.16720	0.07700	0.12060	0.14850	0.14410	0.13773	0.03102
2LWK	3	0.31200	0.13070	0.18370	0.21710	0.28450	0.22843	0.06598
2LWK	4	0.32450	0.21830	0.22830	0.25080	0.29110	0.25673	0.03980
2LWK	5	0.25910	0.23040	0.11440	0.18300	0.19540	0.20293	0.04897
3Q50	crystal	0.03670	0.04380	0.03960	0.04140	0.04590	0.04160	0.00320
3Q50	1	0.05540	0.05280	0.04510	0.05180	0.04870	0.05110	0.00355
3Q50	2	0.03560	0.02380	0.02030	0.02060	0.03000	0.02480	0.00591
3Q50	3	0.19010	0.11600	0.12100	0.18840	0.24830	0.16650	0.04930
3Q50	4	0.09840	0.07640	0.09770	0.08150	0.09510	0.09143	0.00909
3Q50	5	0.06270	0.04340	0.05020	0.04410	0.05090	0.04840	0.00693
5BJO	crystal	0.02850	0.03840	0.07830	0.02800	0.02840	0.03177	0.01939
5BJO	1	0.03830	0.05980	0.03270	0.02690	0.37550	0.04360	0.13489
5BJO	2	0.18070	0.17780	0.12750	0.15610	0.15960	0.16450	0.01906
5BJO	3	0.13330	0.09210	0.13230	0.11740	0.18780	0.12767	0.03137
5BJO	4	0.05010	0.04560	0.03250	0.04380	0.05190	0.04650	0.00680
5BJO	5	0.18540	0.17780	0.25500	0.24130	0.28270	0.22723	0.04058
6EIU	crystal	0.03220	0.02610	0.04200	0.03520	0.06180	0.03647	0.01229
6EIU	1	0.03120	0.03810	0.04400	0.08800	0.03800	0.04003	0.02048
6EIU	2	0.07670	0.13790	0.07840	0.08360	0.13630	0.09943	0.02828
6EIU	3	0.03880	0.04360	0.05380	0.02920	0.03560	0.03933	0.00825
6EIU	4	0.06950	0.12540	0.07430	0.08380	0.13860	0.09450	0.02819
6EIU	5	0.06190	0.05220	0.04590	0.06050	0.06560	0.05820	0.00716

Table S1. This table summarizes results for TTMD simulations carried out under the protocol 2 simulation and analysis conditions. For each investigated system, the individual IFF coefficient for each TTMD replicate and the average IFF value are reported. The closest value to the mean is highlighted.

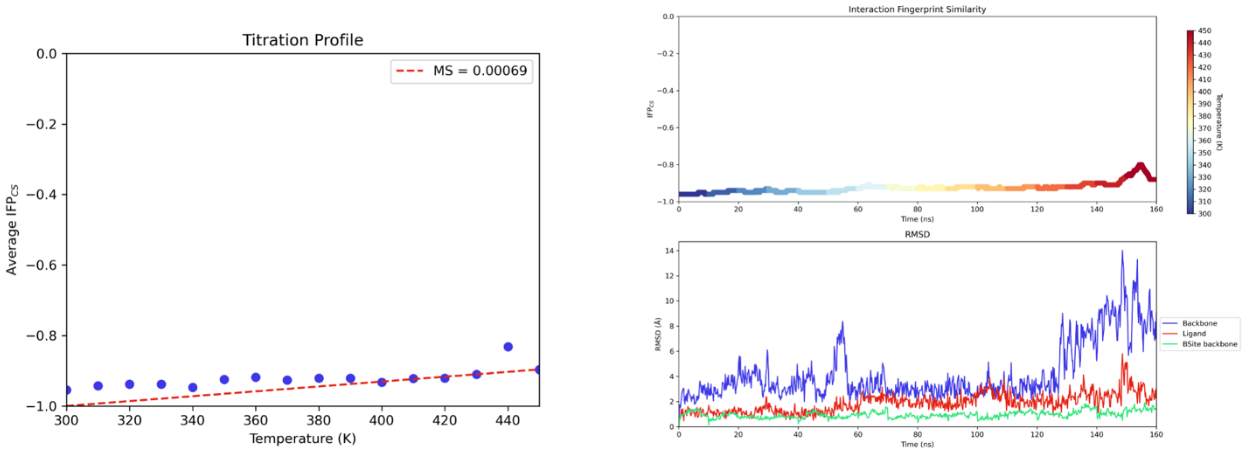

Figure S1. Titration Profile and Titration Timeline graphs of 5BJO selected replica of the best-performing docking pose (pose 1).

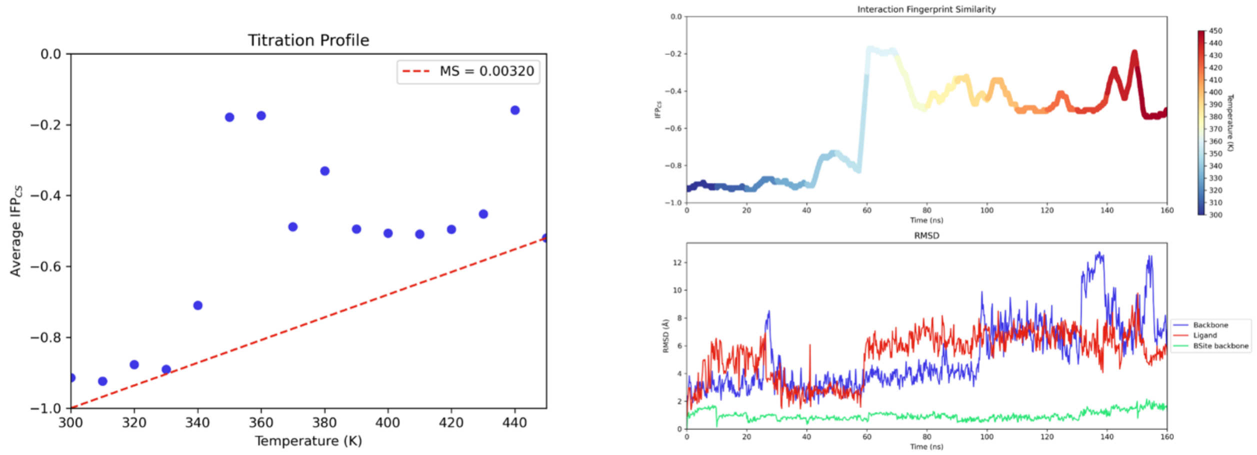

Figure S2. Titration Profile and Titration Timeline graphs of 5BJO selected replica of worst performing docking pose (pose 5).

All trajectories presented in the article can be found at https://doi.org/10.5281/zenodo.8345266.
